# Supplementary figures and images for: Promoting axon regeneration by inhibiting RNA N6-methyladenosine demethylase ALKBH5
Source: eLife. 2023 Aug 3;12:e85309. doi: 10.7554/eLife.85309 (PMC10400074; doi:10.7554/eLife.85309)

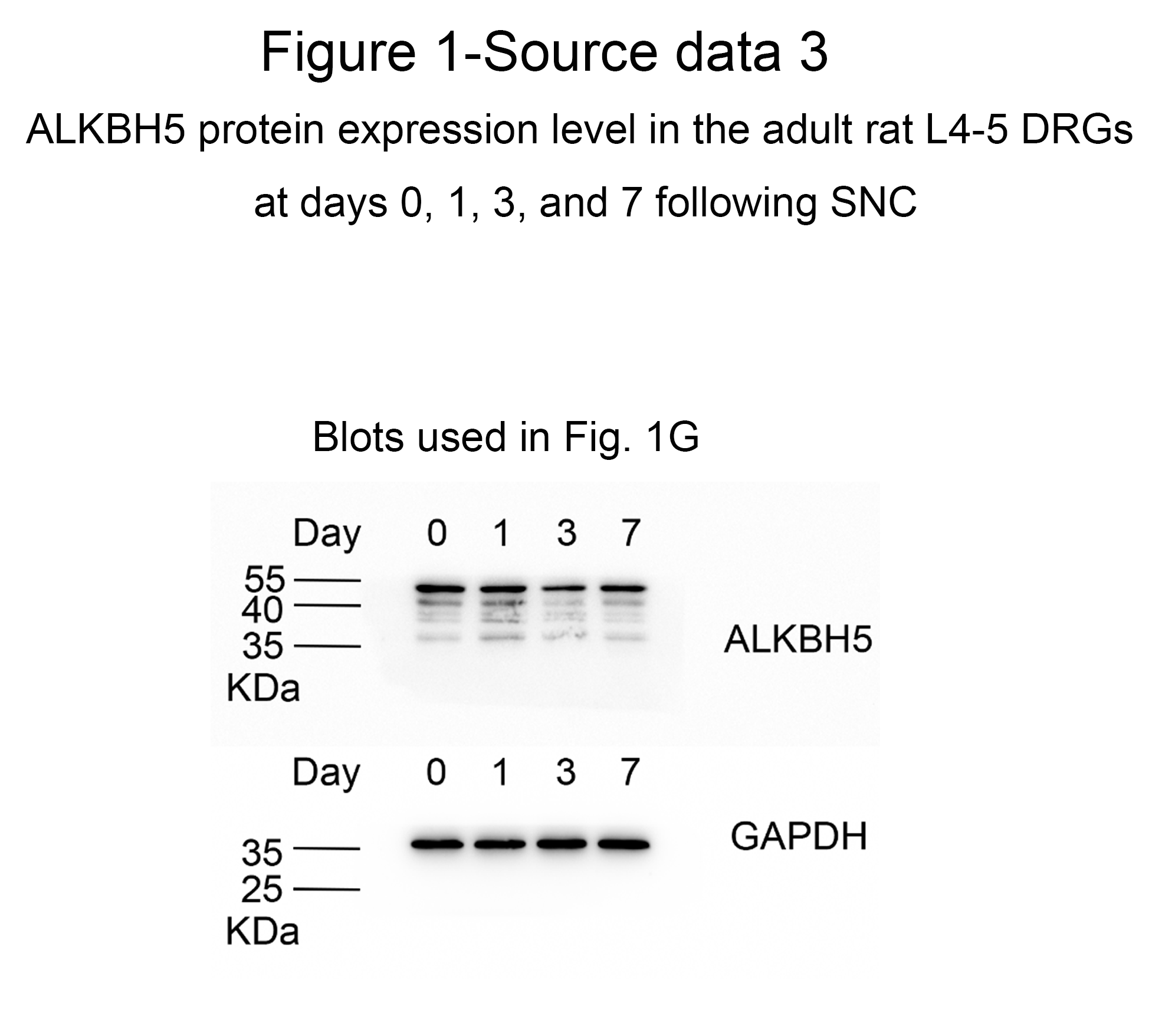

Supplement: Figure 1—source data 3. [file elife-85309-fig1-data3.zip › Figure 1-Source data 3/Figure 1-Source data 3.tif]

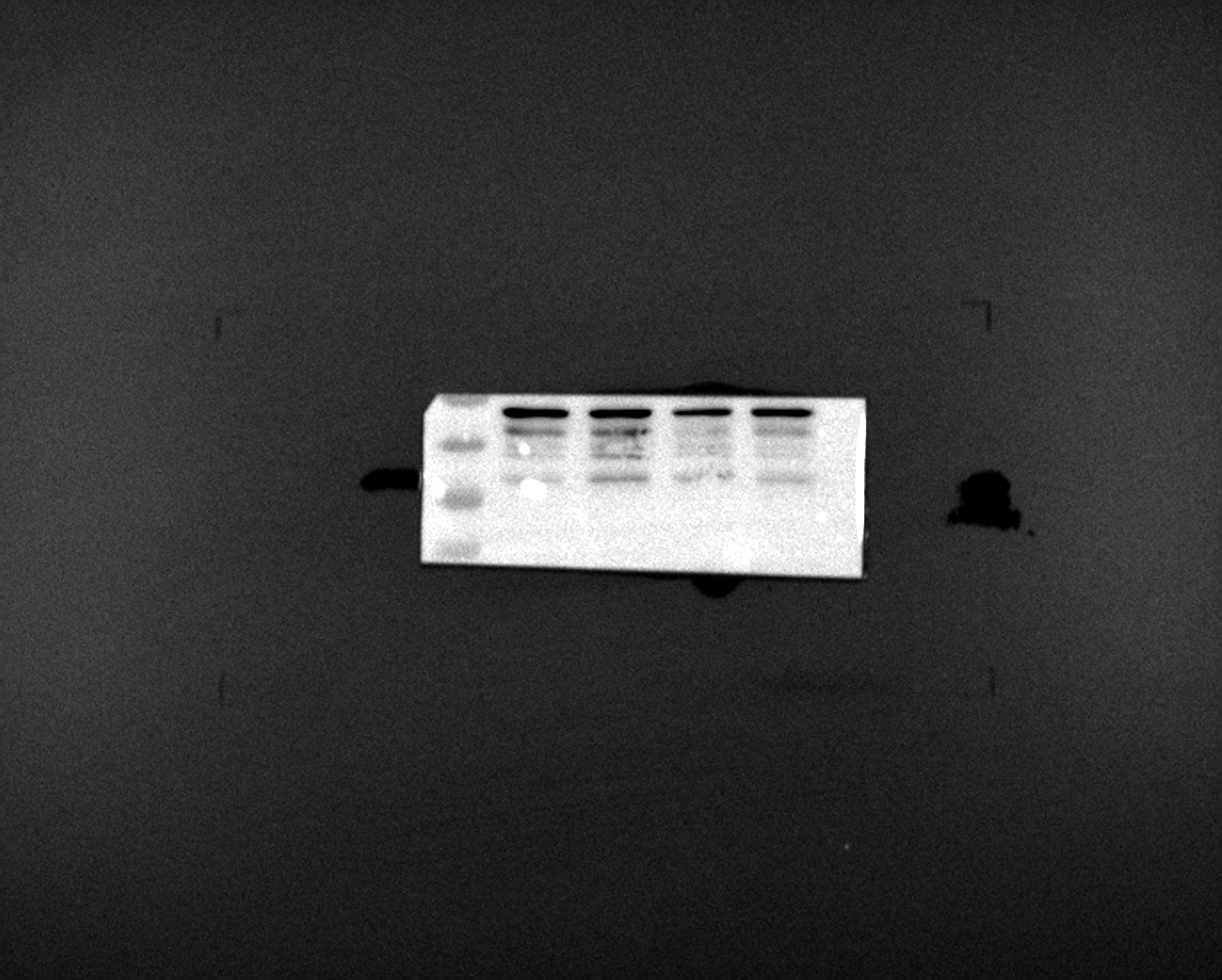

Supplement: Figure 1—source data 3. [file elife-85309-fig1-data3.zip › Figure 1-Source data 3/unedited/ALKBH5-merge.Tif]

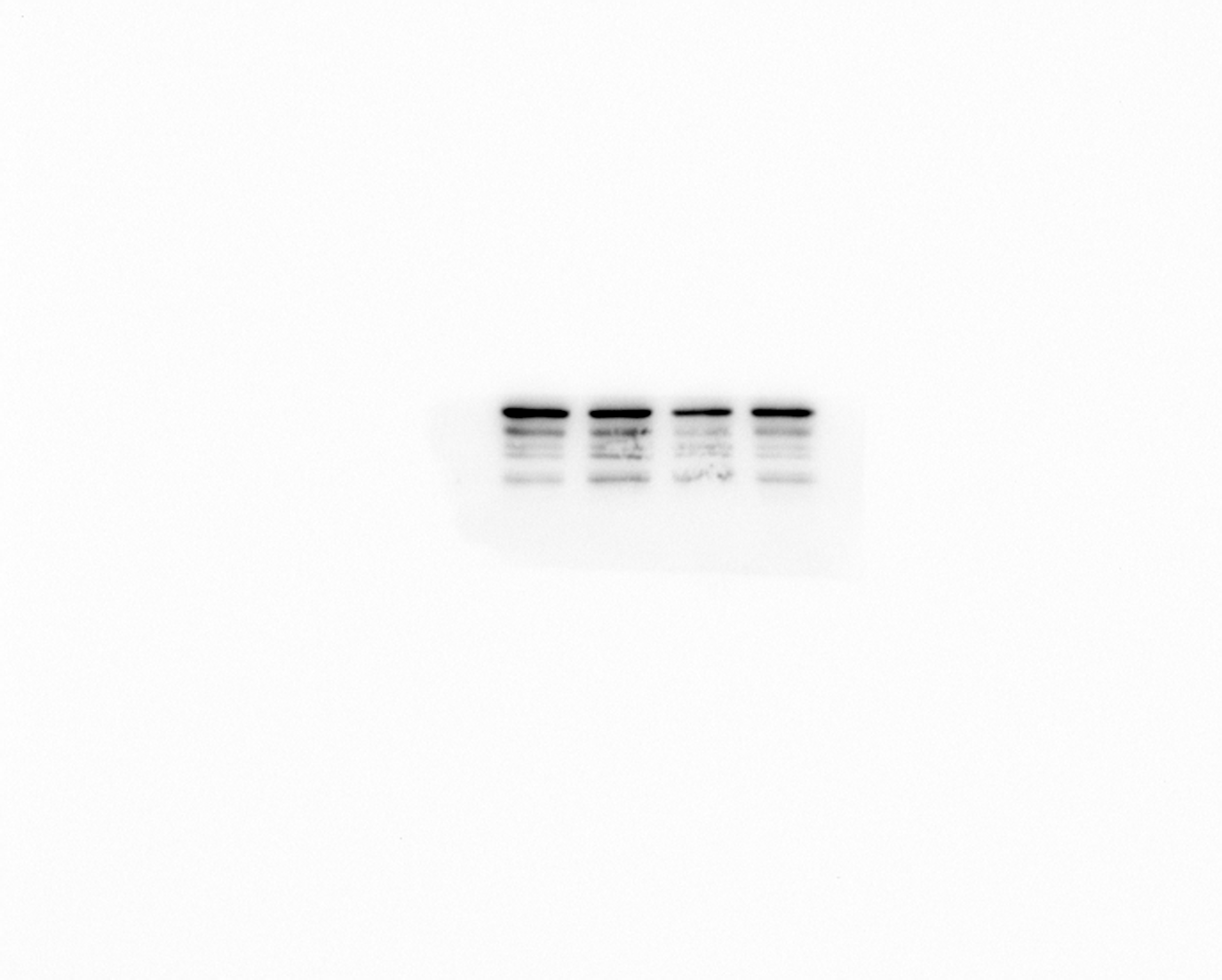

Supplement: Figure 1—source data 3. [file elife-85309-fig1-data3.zip › Figure 1-Source data 3/unedited/ALKBH5.Tif]

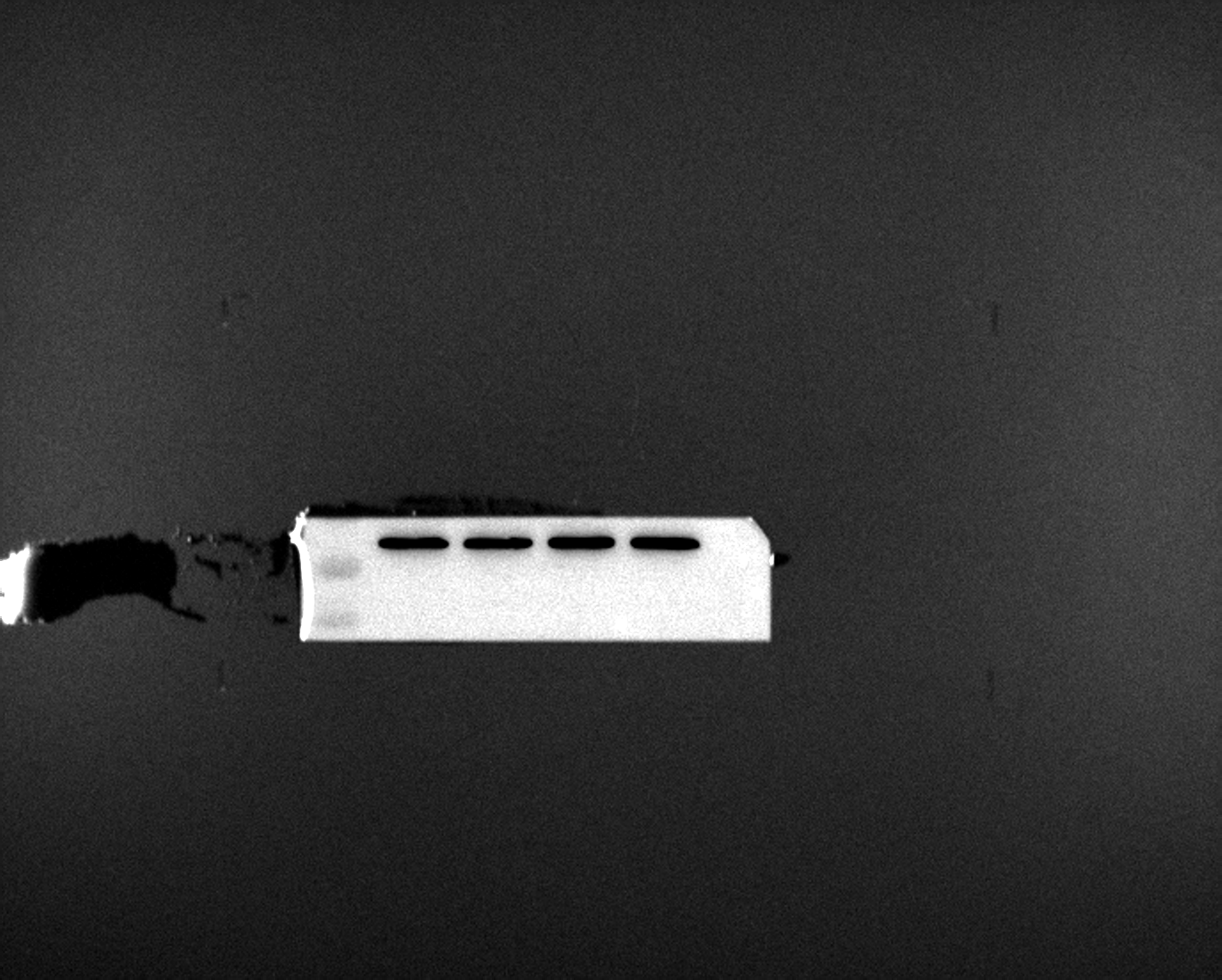

Supplement: Figure 1—source data 3. [file elife-85309-fig1-data3.zip › Figure 1-Source data 3/unedited/GAPDH-merge.Tif]

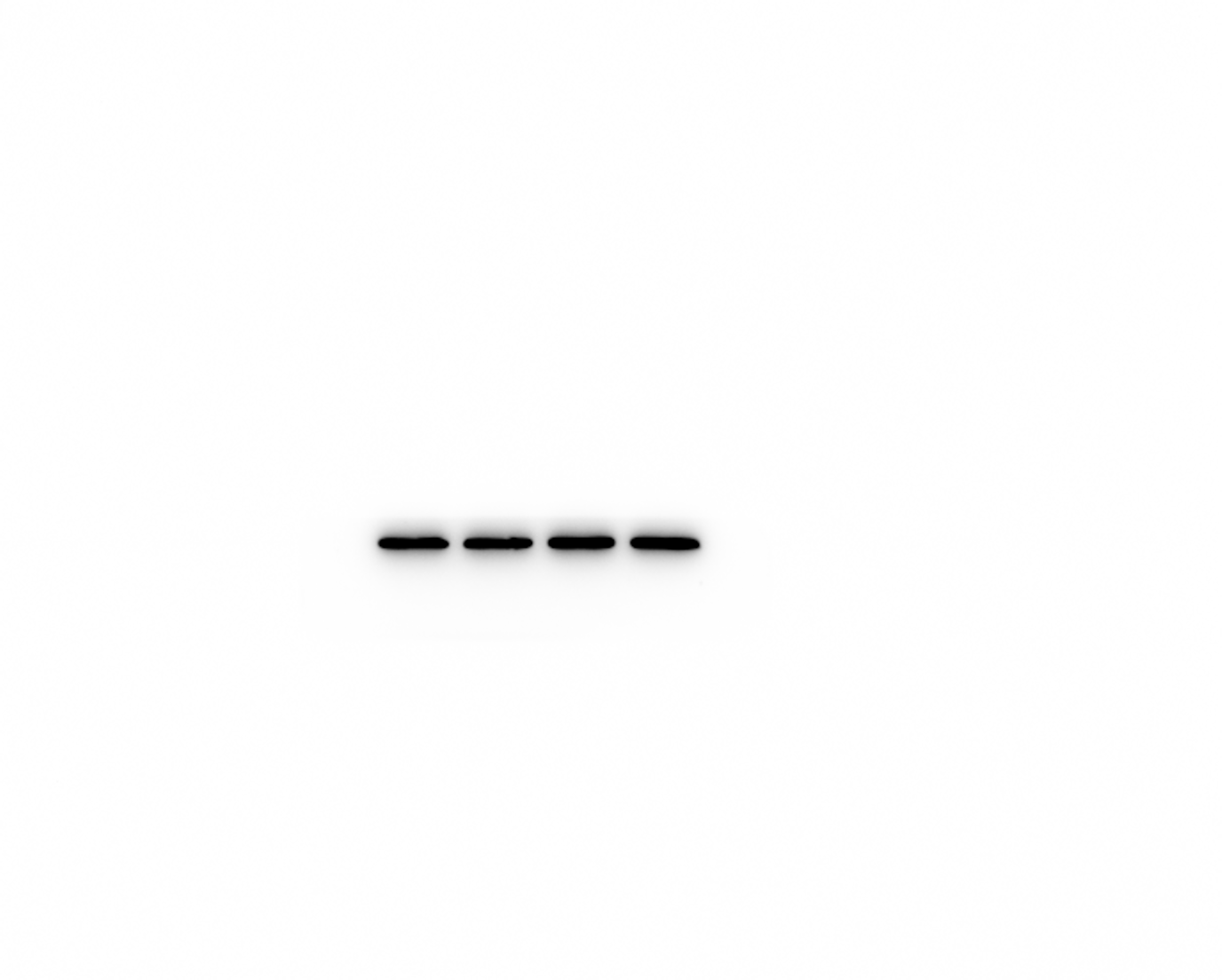

Supplement: Figure 1—source data 3. [file elife-85309-fig1-data3.zip › Figure 1-Source data 3/unedited/GAPDH.Tif]

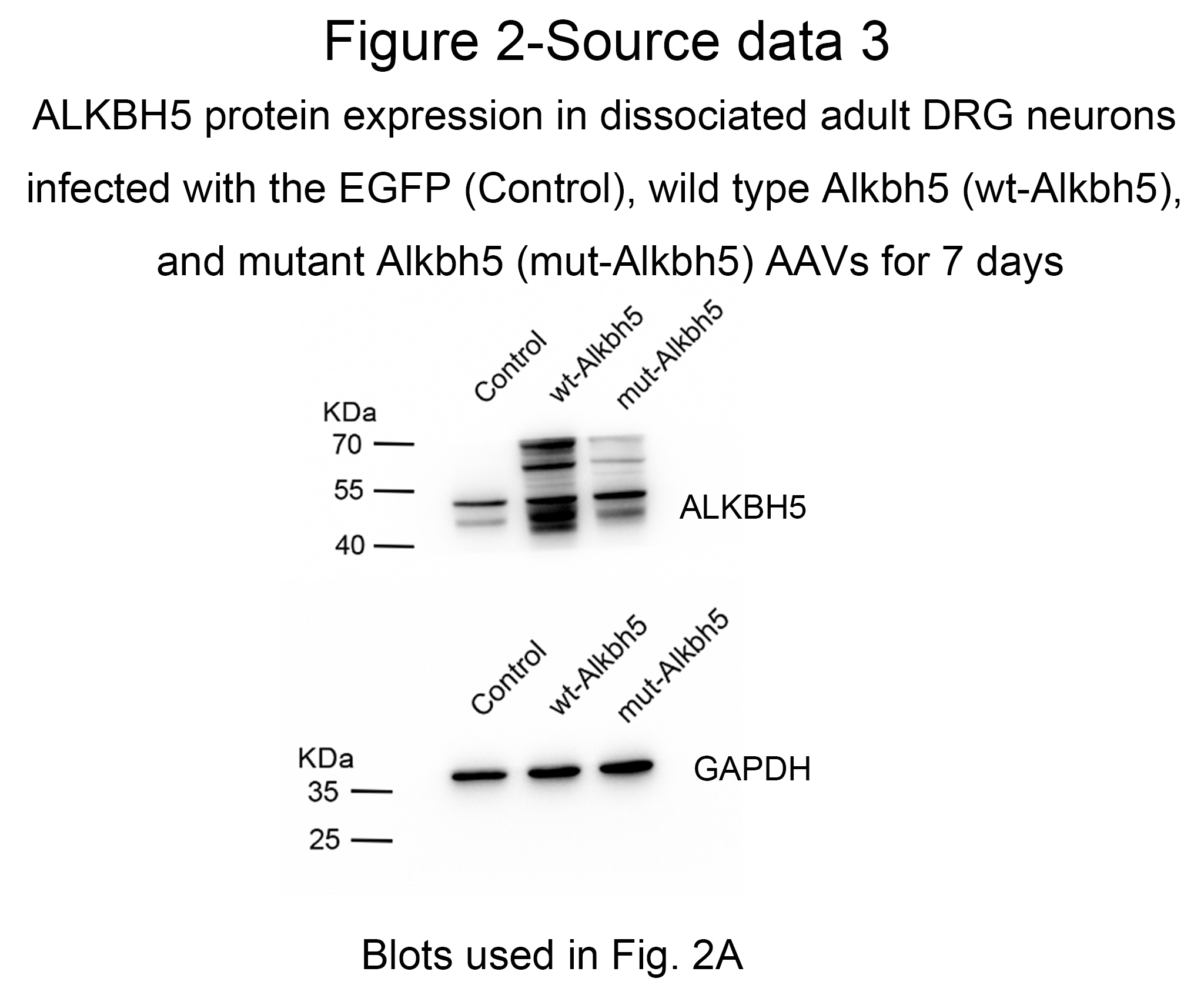

Supplement: Figure 2—source data 3. [file elife-85309-fig2-data3.zip › Figure 2-Source data 3/Figure 2-Source data 3.tif]

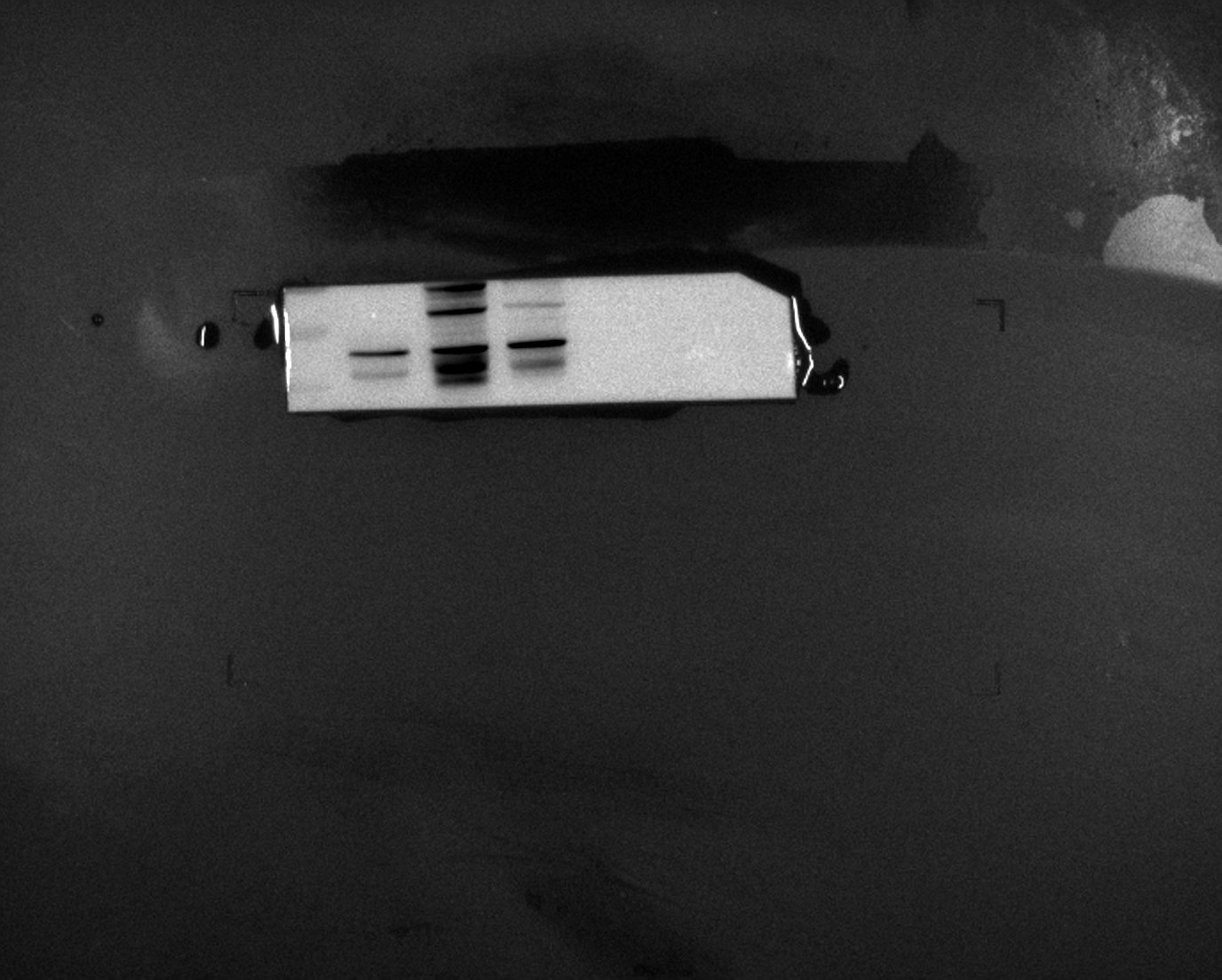

Supplement: Figure 2—source data 3. [file elife-85309-fig2-data3.zip › Figure 2-Source data 3/unedited/ALKBH5-merge.tif]

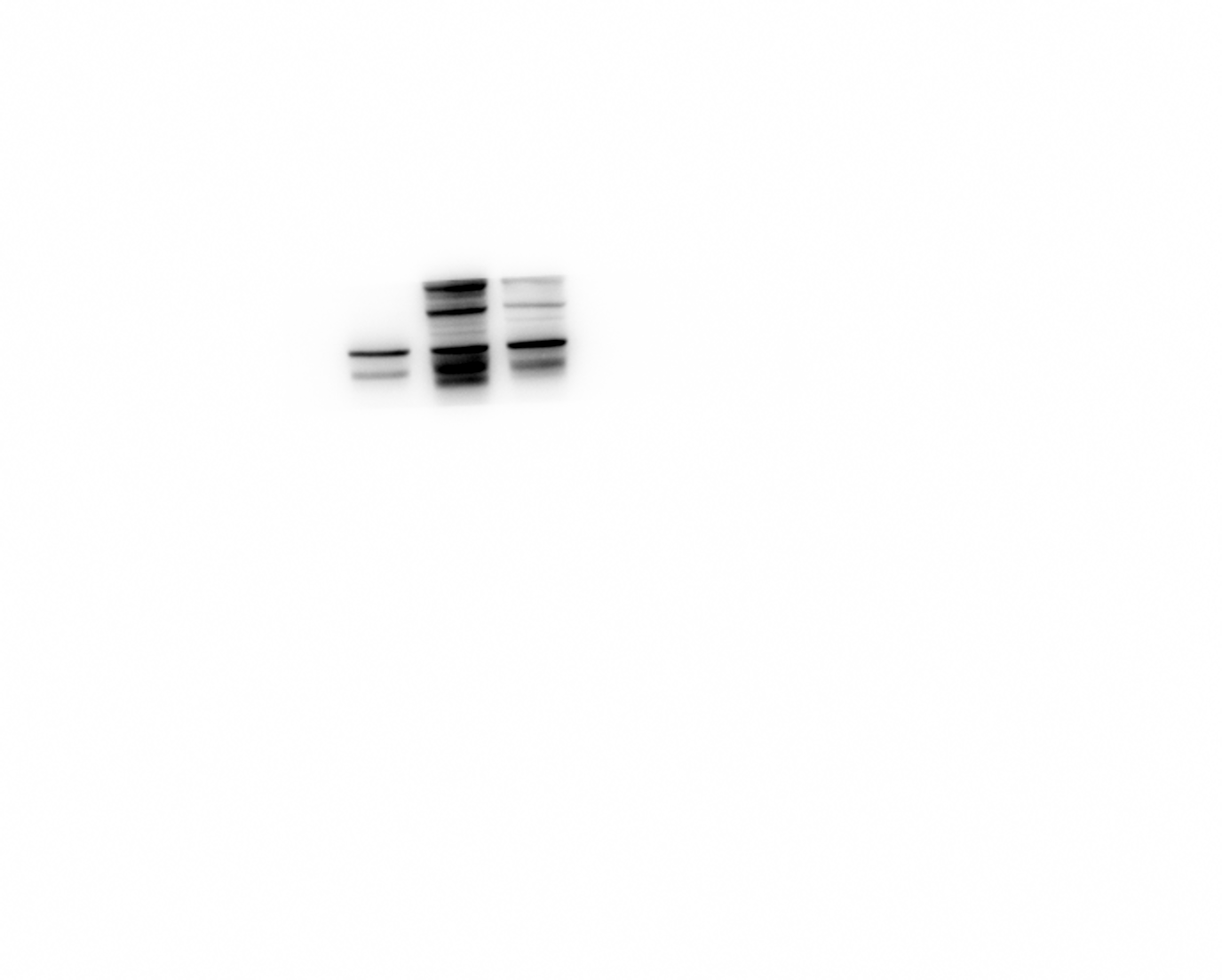

Supplement: Figure 2—source data 3. [file elife-85309-fig2-data3.zip › Figure 2-Source data 3/unedited/ALKBH5.Tif]

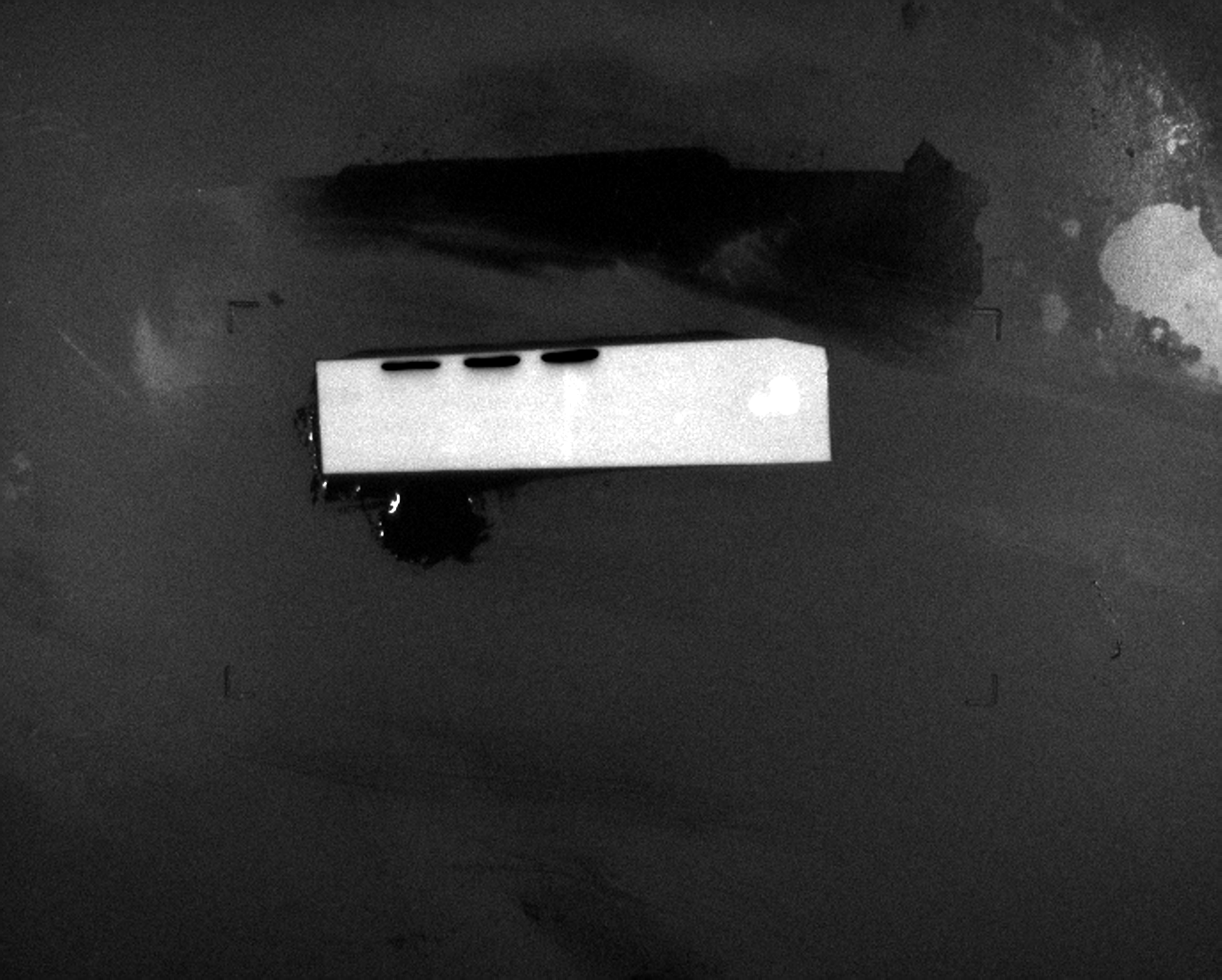

Supplement: Figure 2—source data 3. [file elife-85309-fig2-data3.zip › Figure 2-Source data 3/unedited/GAPDH-merge.Tif]

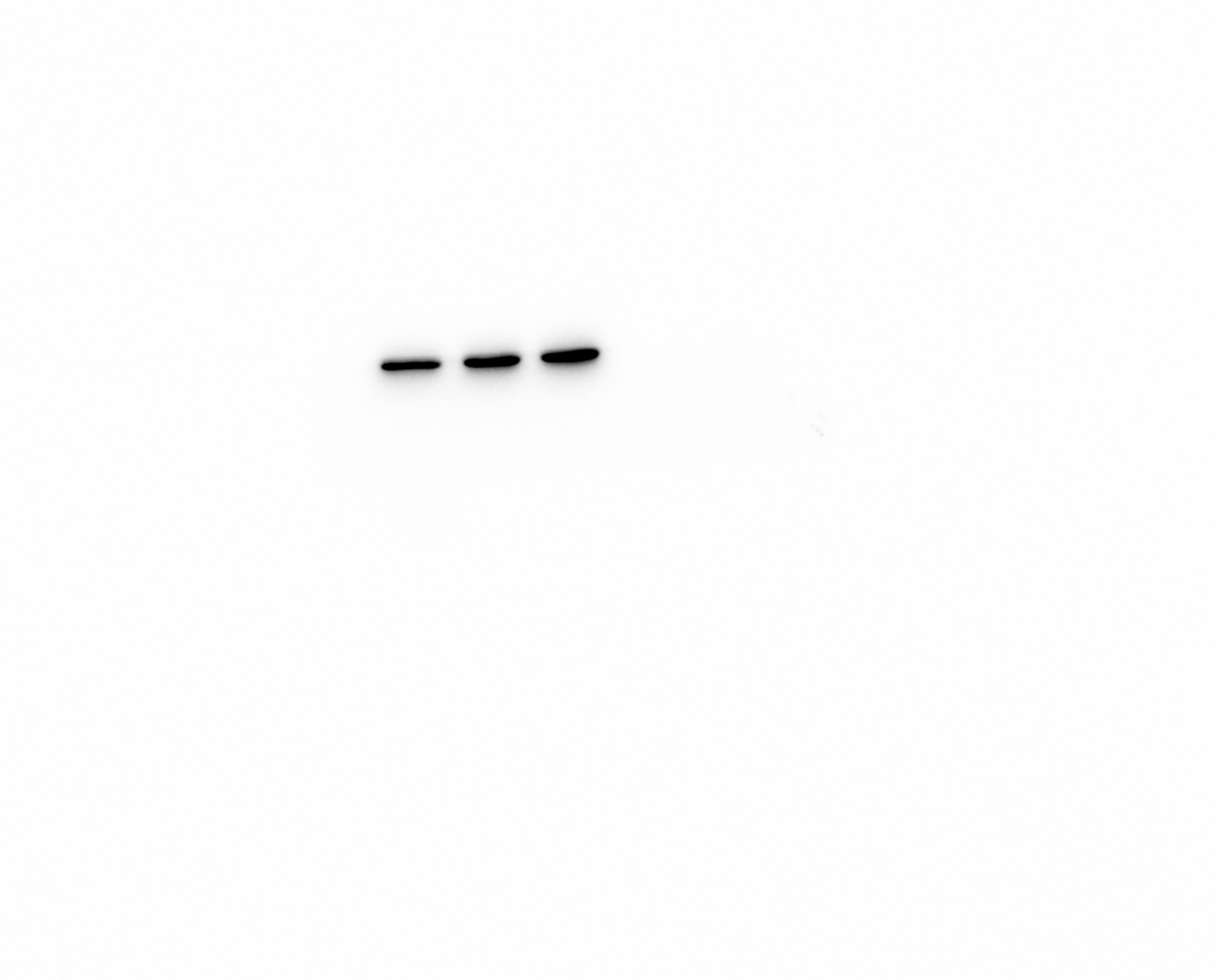

Supplement: Figure 2—source data 3. [file elife-85309-fig2-data3.zip › Figure 2-Source data 3/unedited/GAPDH.Tif]

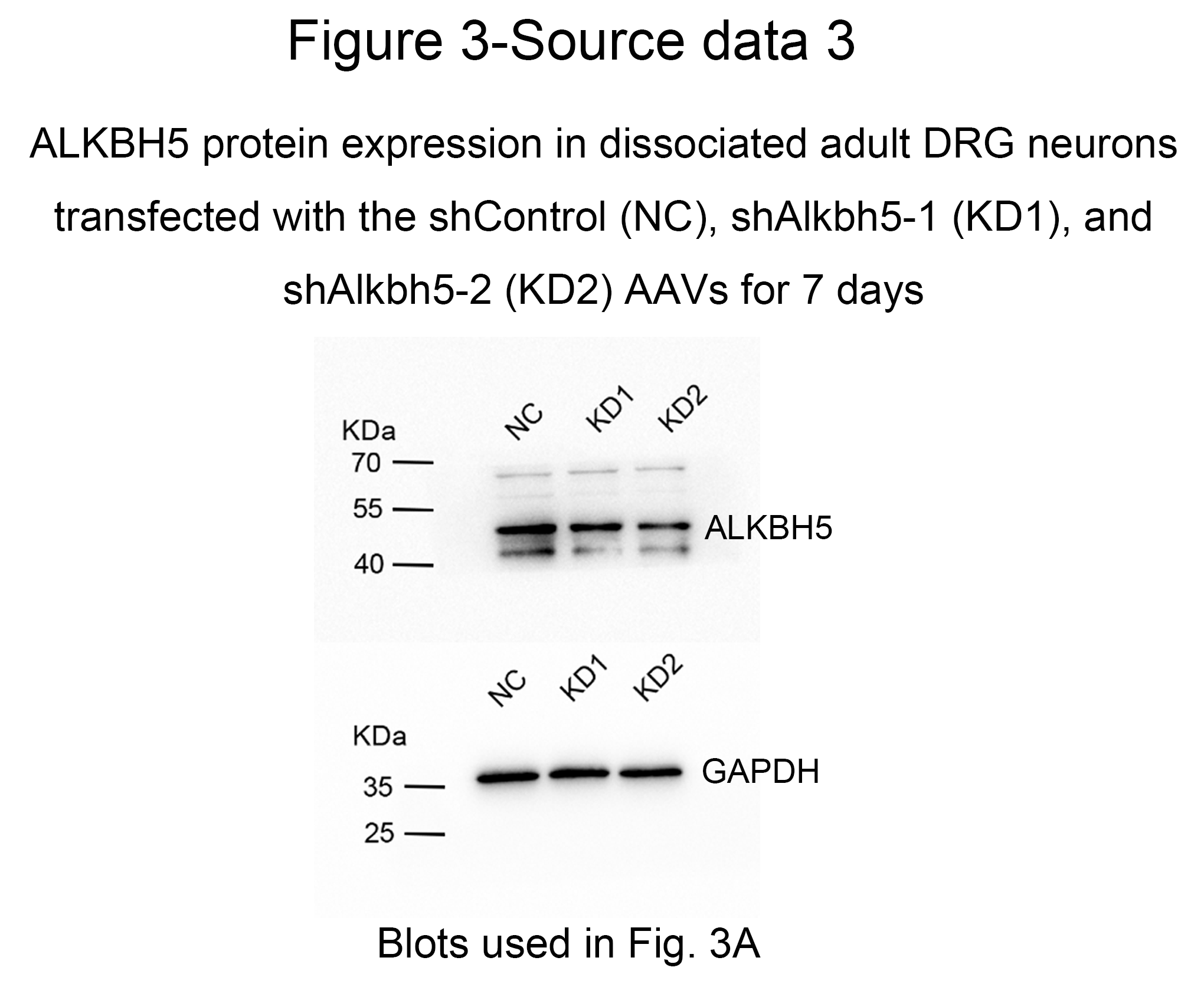

Supplement: Figure 3—source data 3. [file elife-85309-fig3-data3.zip › Figure 3-Source data 3/Figure 3-Source data 3.tif]

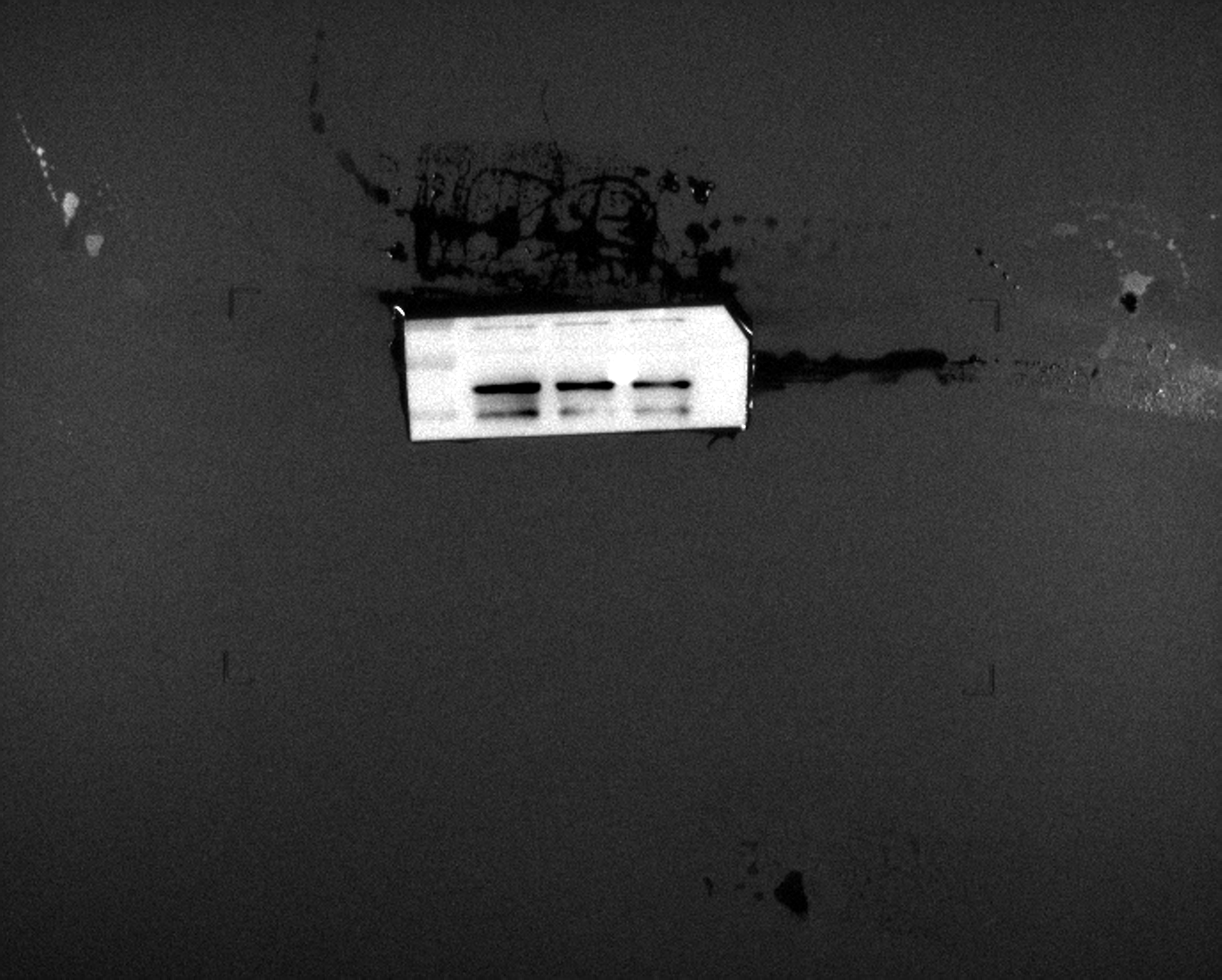

Supplement: Figure 3—source data 3. [file elife-85309-fig3-data3.zip › Figure 3-Source data 3/unedited/ALKBH5-merge.Tif]

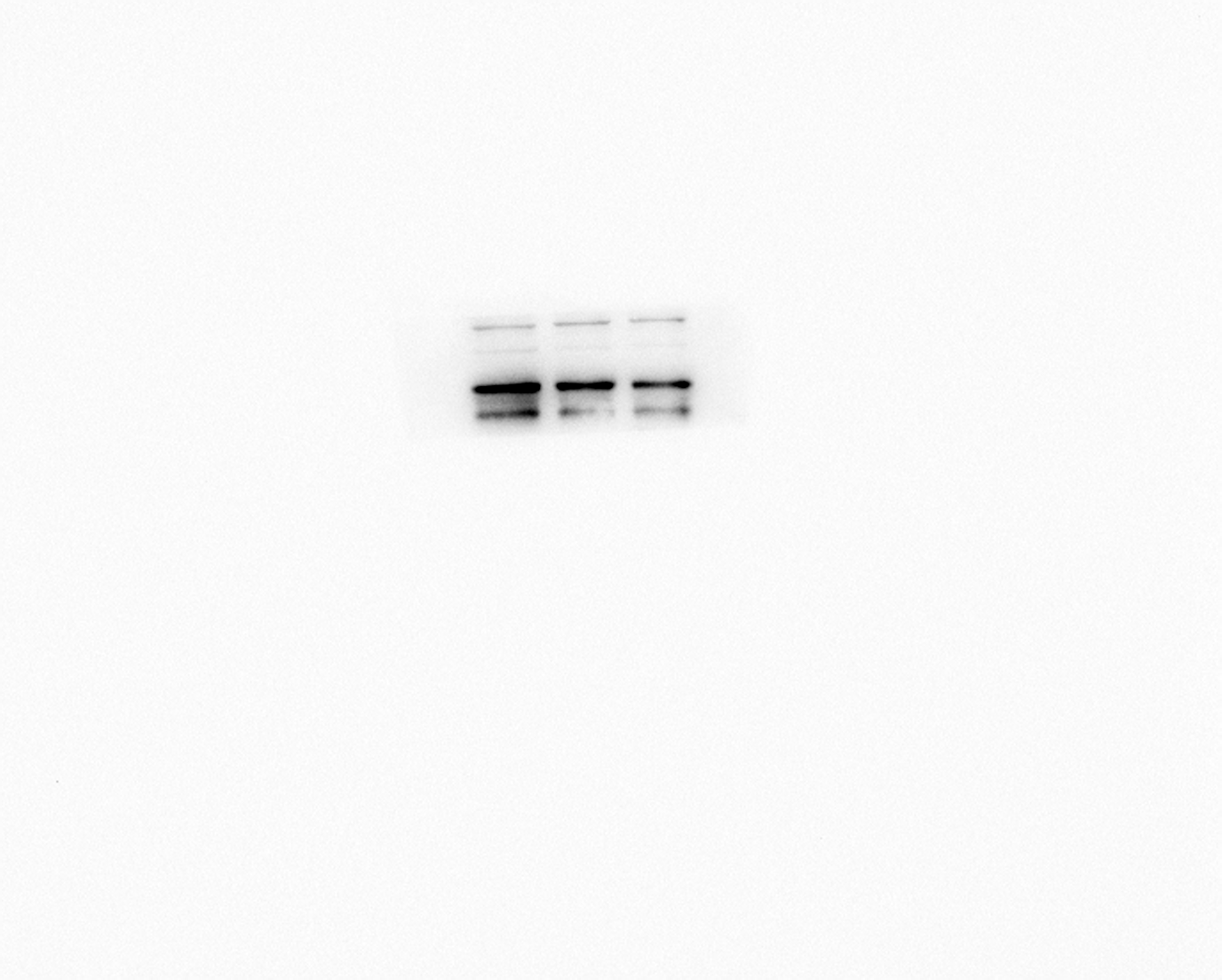

Supplement: Figure 3—source data 3. [file elife-85309-fig3-data3.zip › Figure 3-Source data 3/unedited/ALKBH5.Tif]

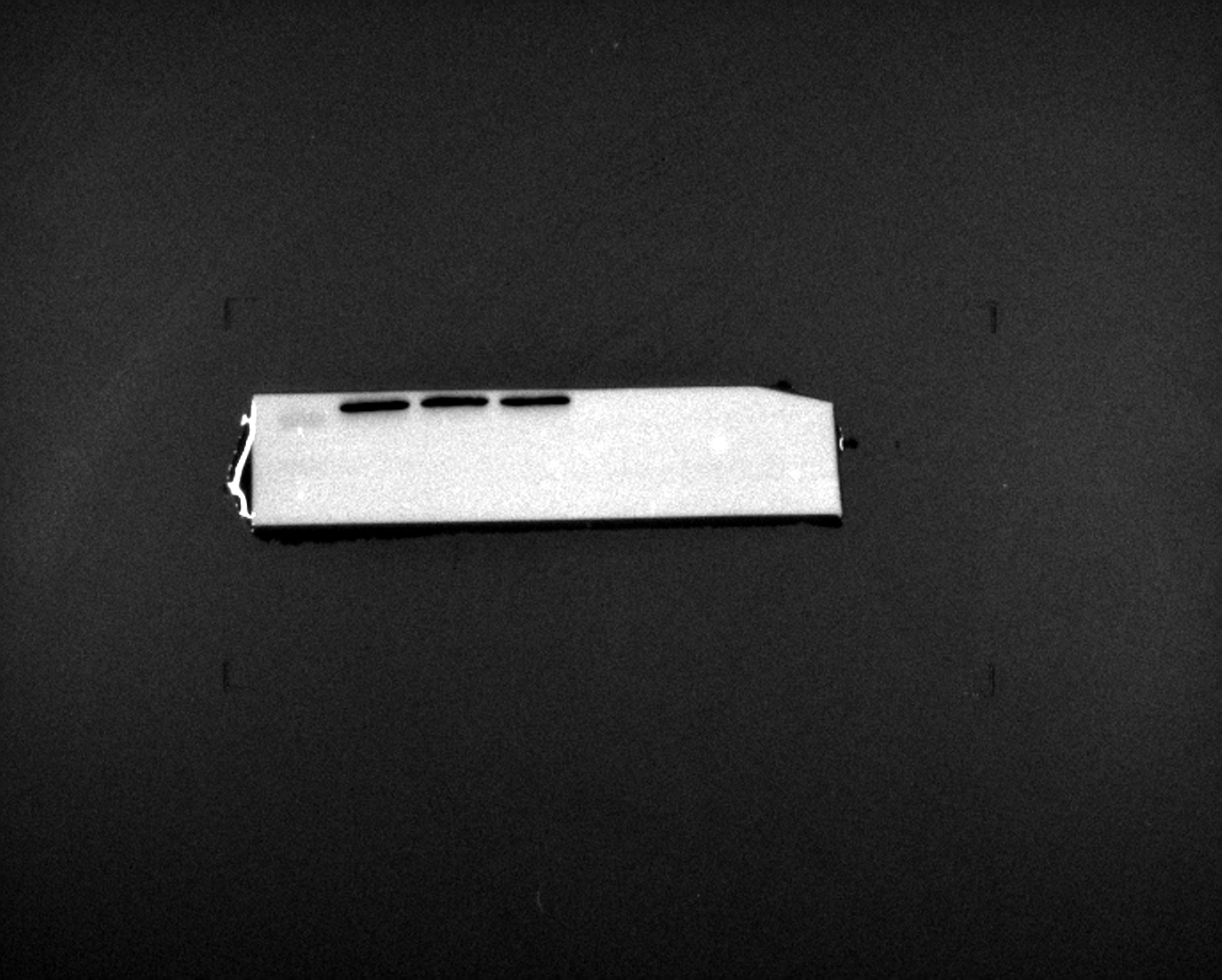

Supplement: Figure 3—source data 3. [file elife-85309-fig3-data3.zip › Figure 3-Source data 3/unedited/GAPDH-merge.Tif]

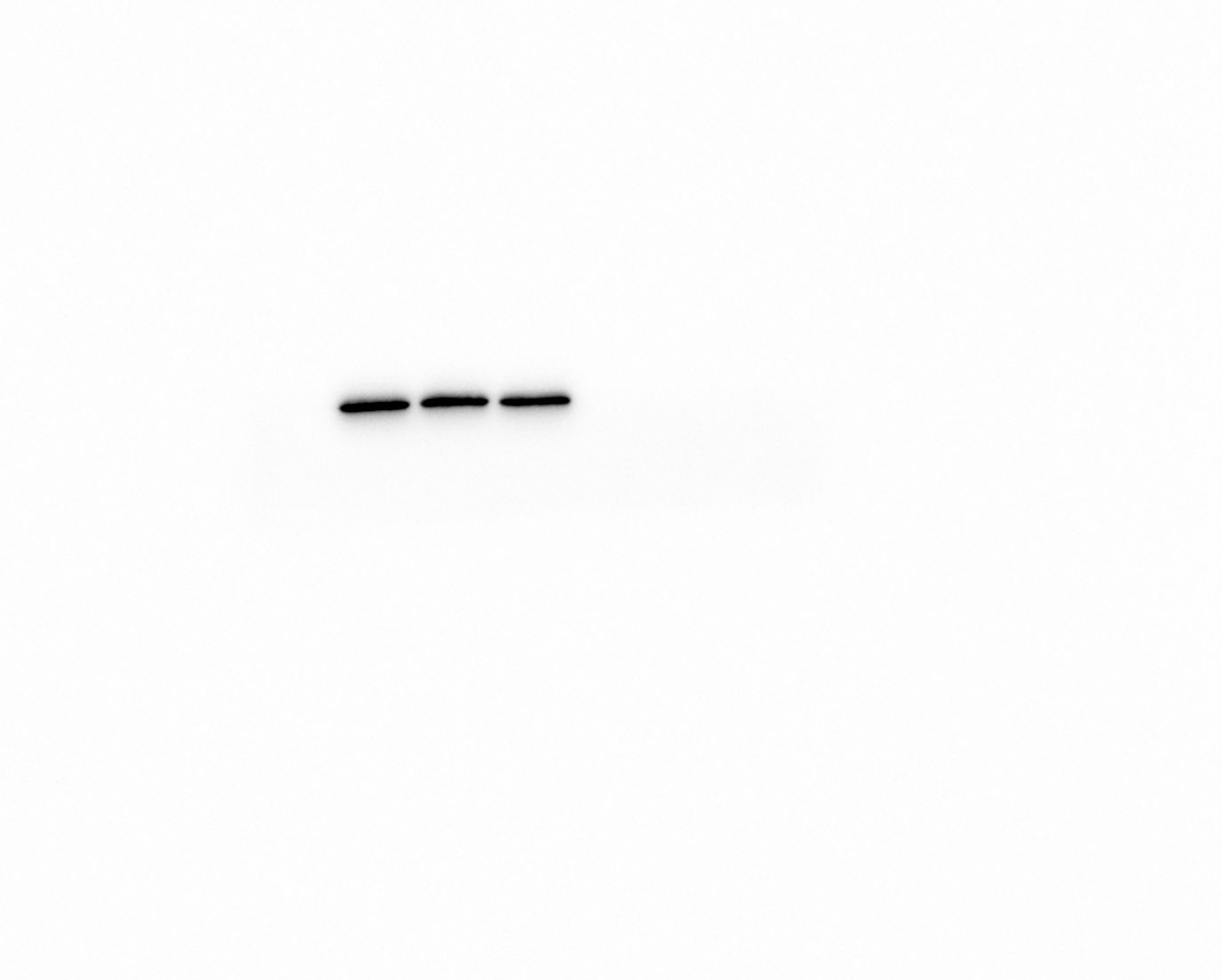

Supplement: Figure 3—source data 3. [file elife-85309-fig3-data3.zip › Figure 3-Source data 3/unedited/GAPDH.Tif]

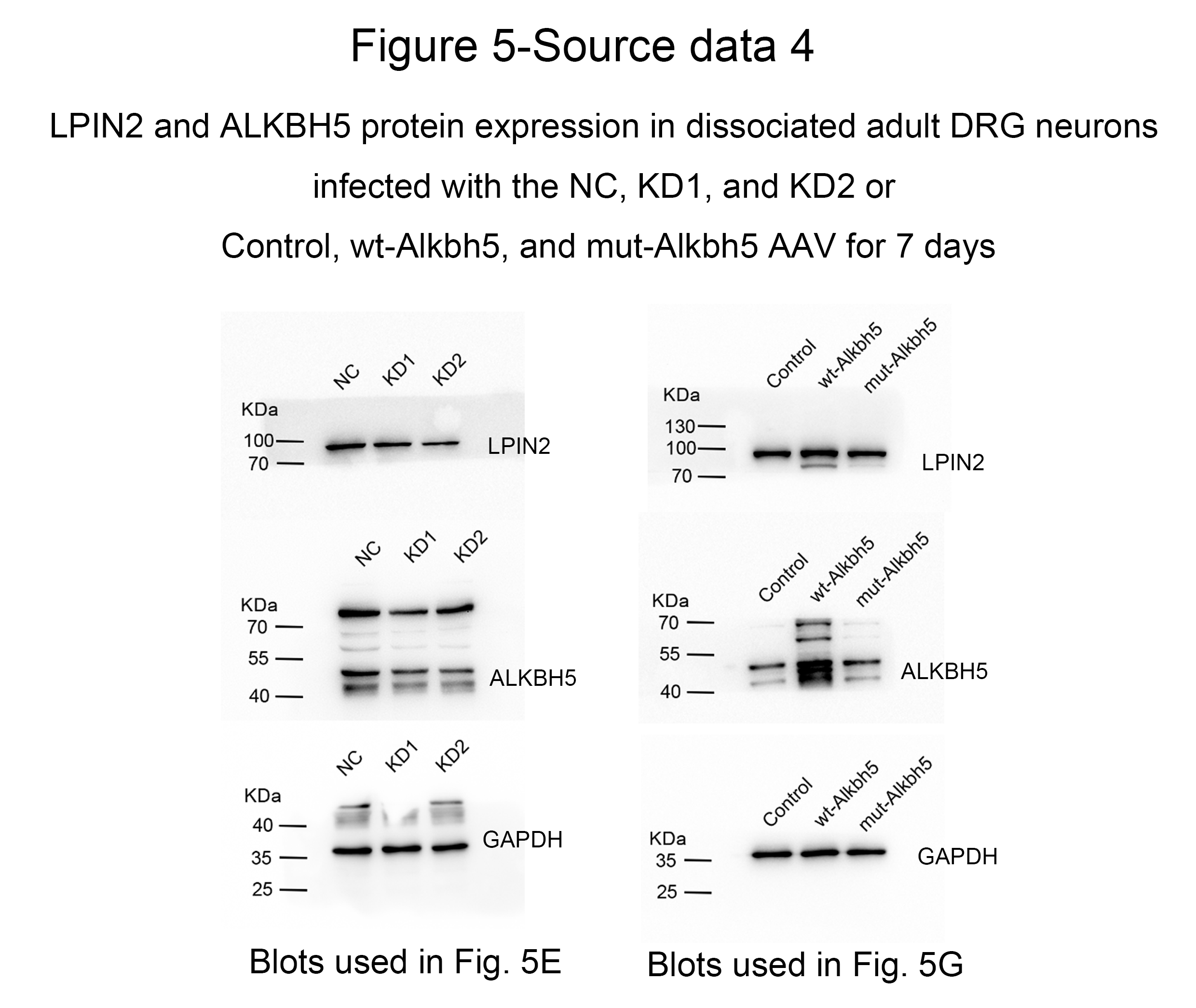

Supplement: Figure 5—source data 4. [file elife-85309-fig5-data4.zip › Figure 5-Source data 4/Figure 5-Source data 4.tif]

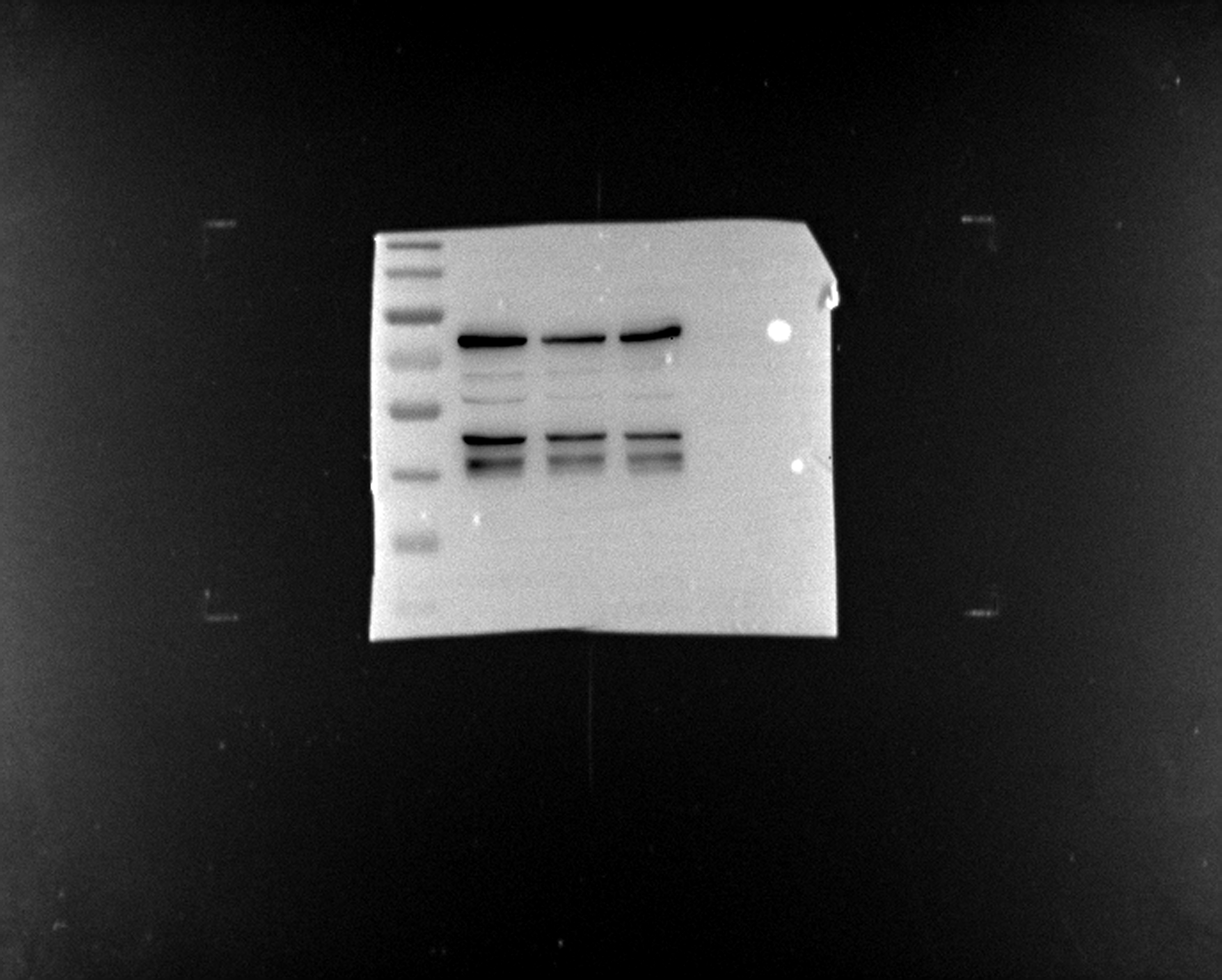

Supplement: Figure 5—source data 4. [file elife-85309-fig5-data4.zip › Figure 5-Source data 4/unedited/E/ALKBH5-merge.tif]

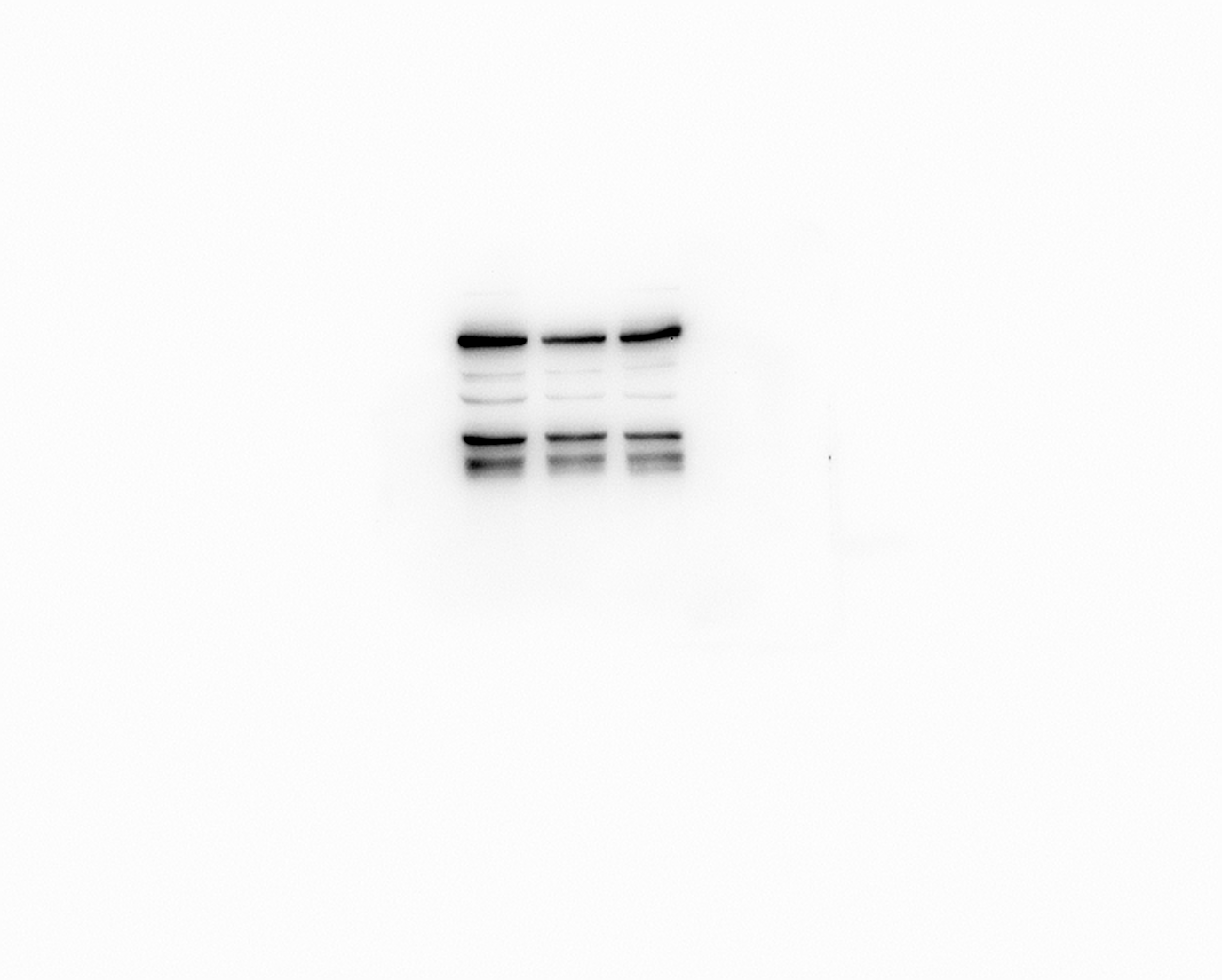

Supplement: Figure 5—source data 4. [file elife-85309-fig5-data4.zip › Figure 5-Source data 4/unedited/E/ALKBH5.tif]

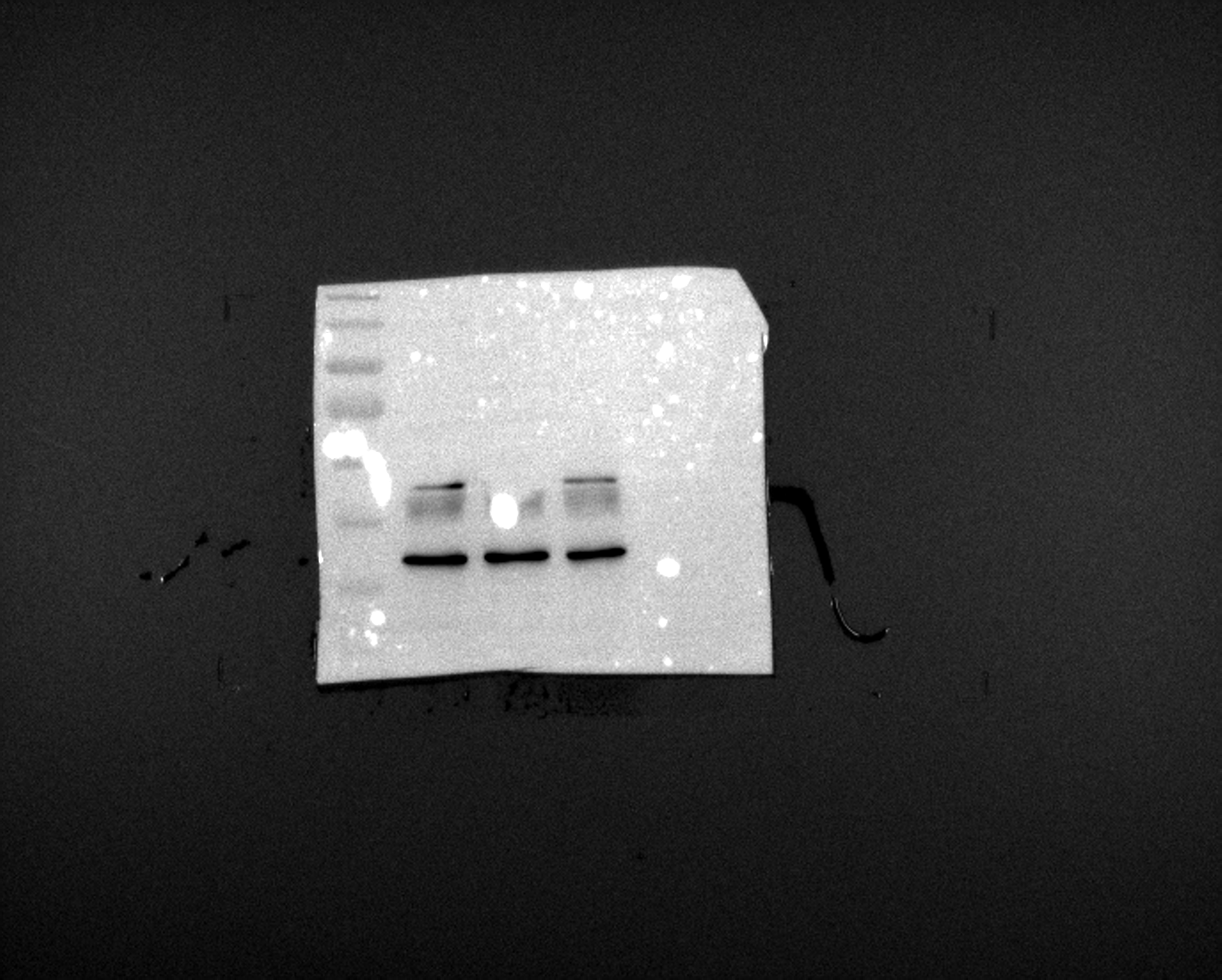

Supplement: Figure 5—source data 4. [file elife-85309-fig5-data4.zip › Figure 5-Source data 4/unedited/E/GAPDH-merge.Tif]

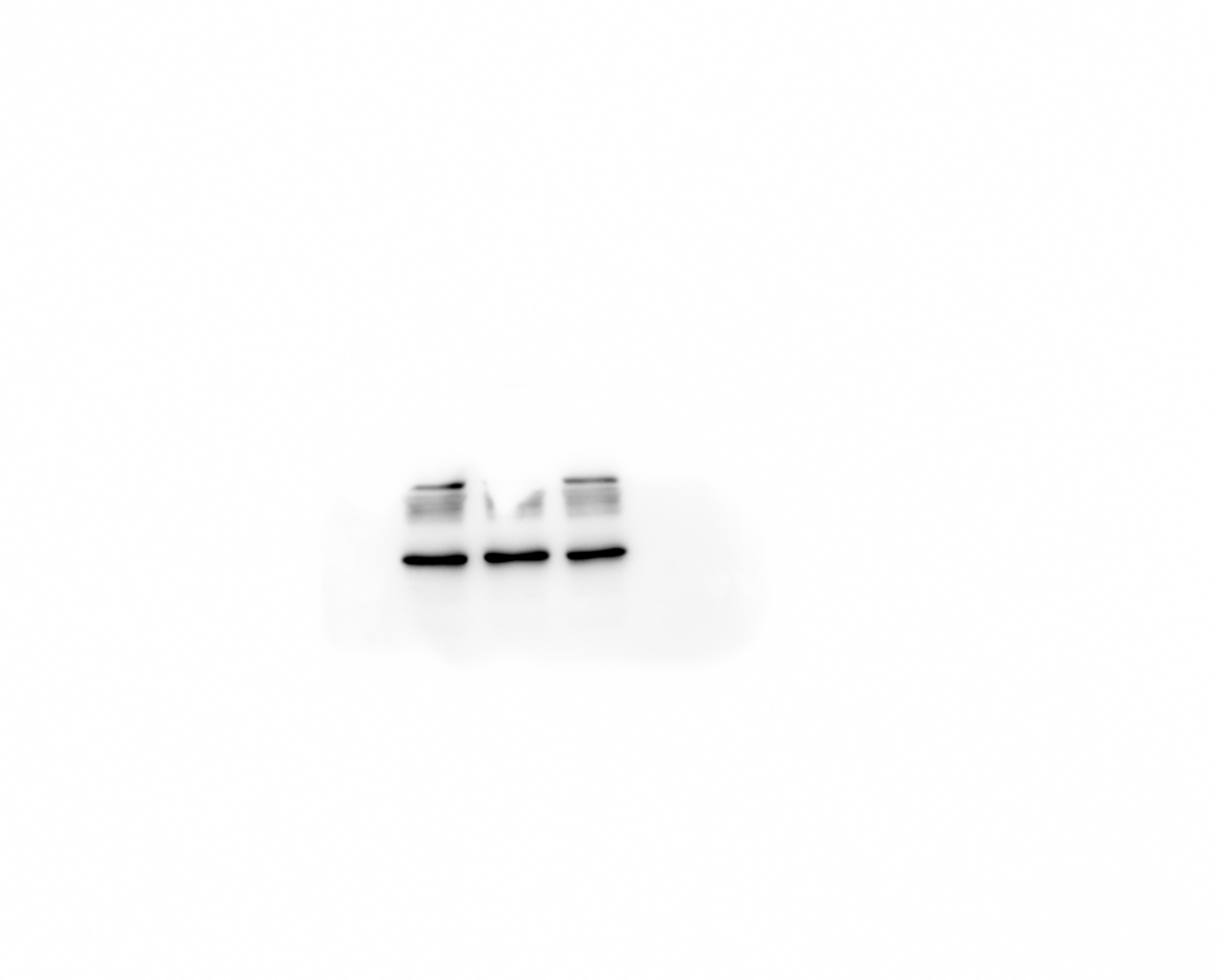

Supplement: Figure 5—source data 4. [file elife-85309-fig5-data4.zip › Figure 5-Source data 4/unedited/E/GAPDH.Tif]

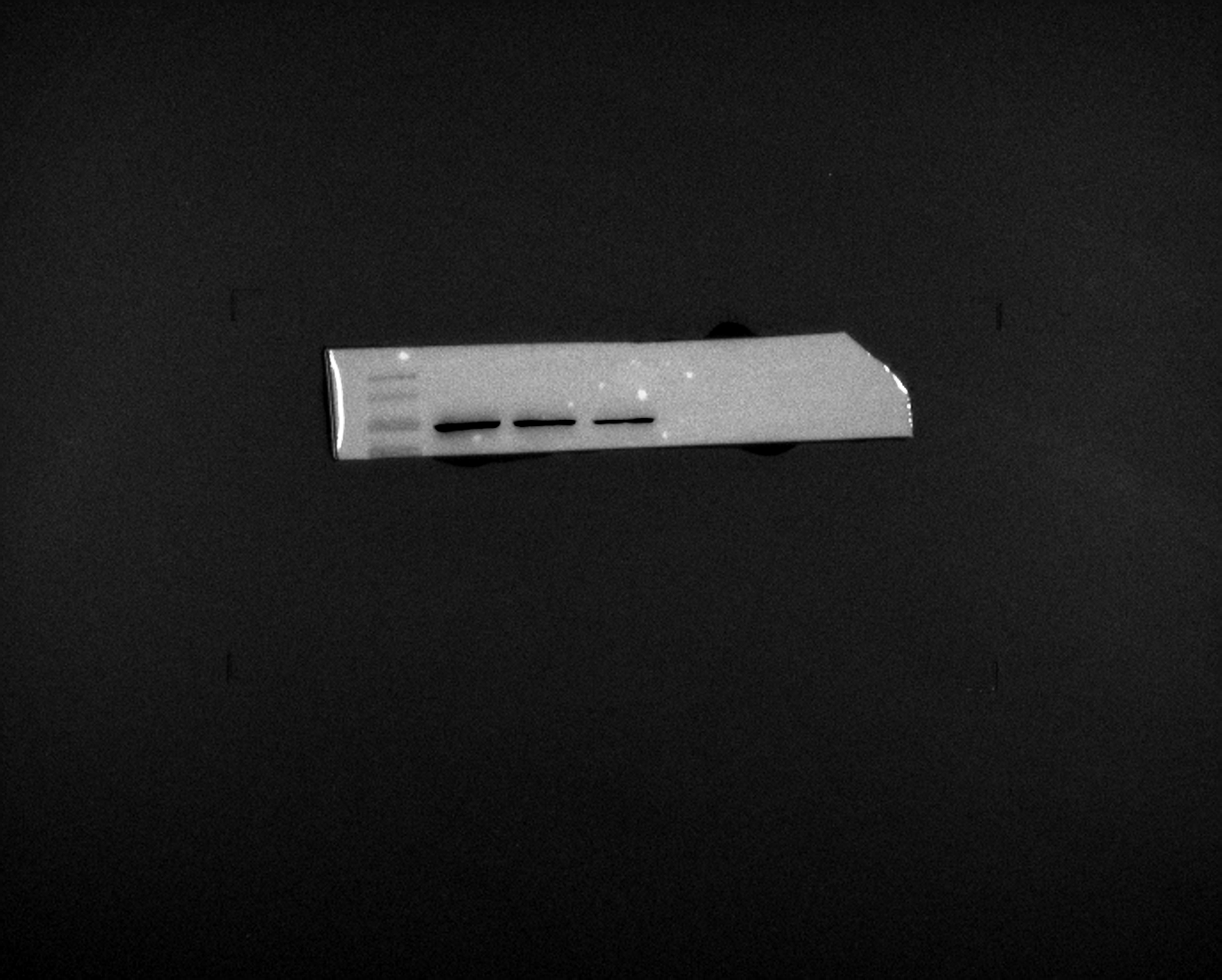

Supplement: Figure 5—source data 4. [file elife-85309-fig5-data4.zip › Figure 5-Source data 4/unedited/E/LIPN2-merge.tif]

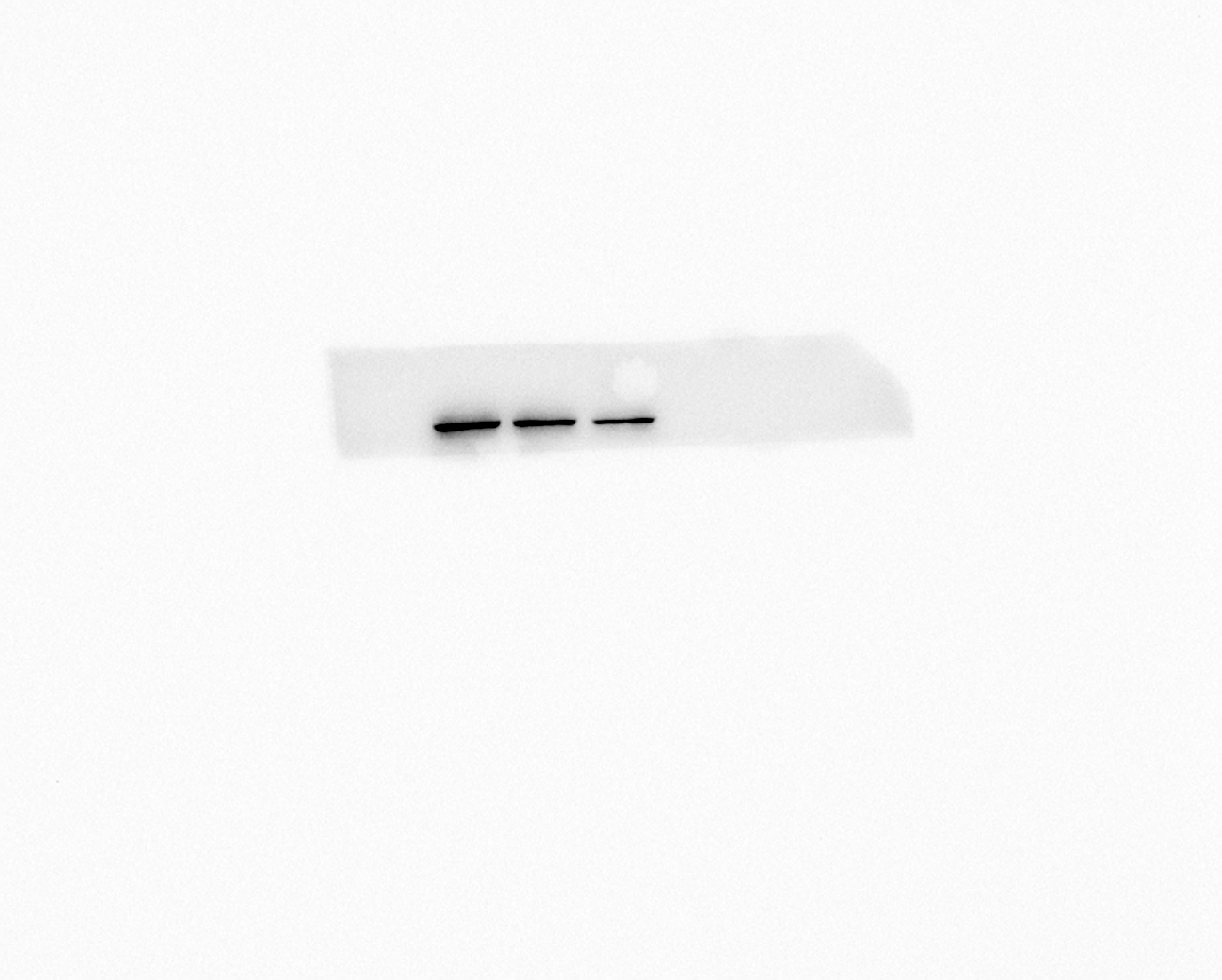

Supplement: Figure 5—source data 4. [file elife-85309-fig5-data4.zip › Figure 5-Source data 4/unedited/E/LIPN2.Tif]

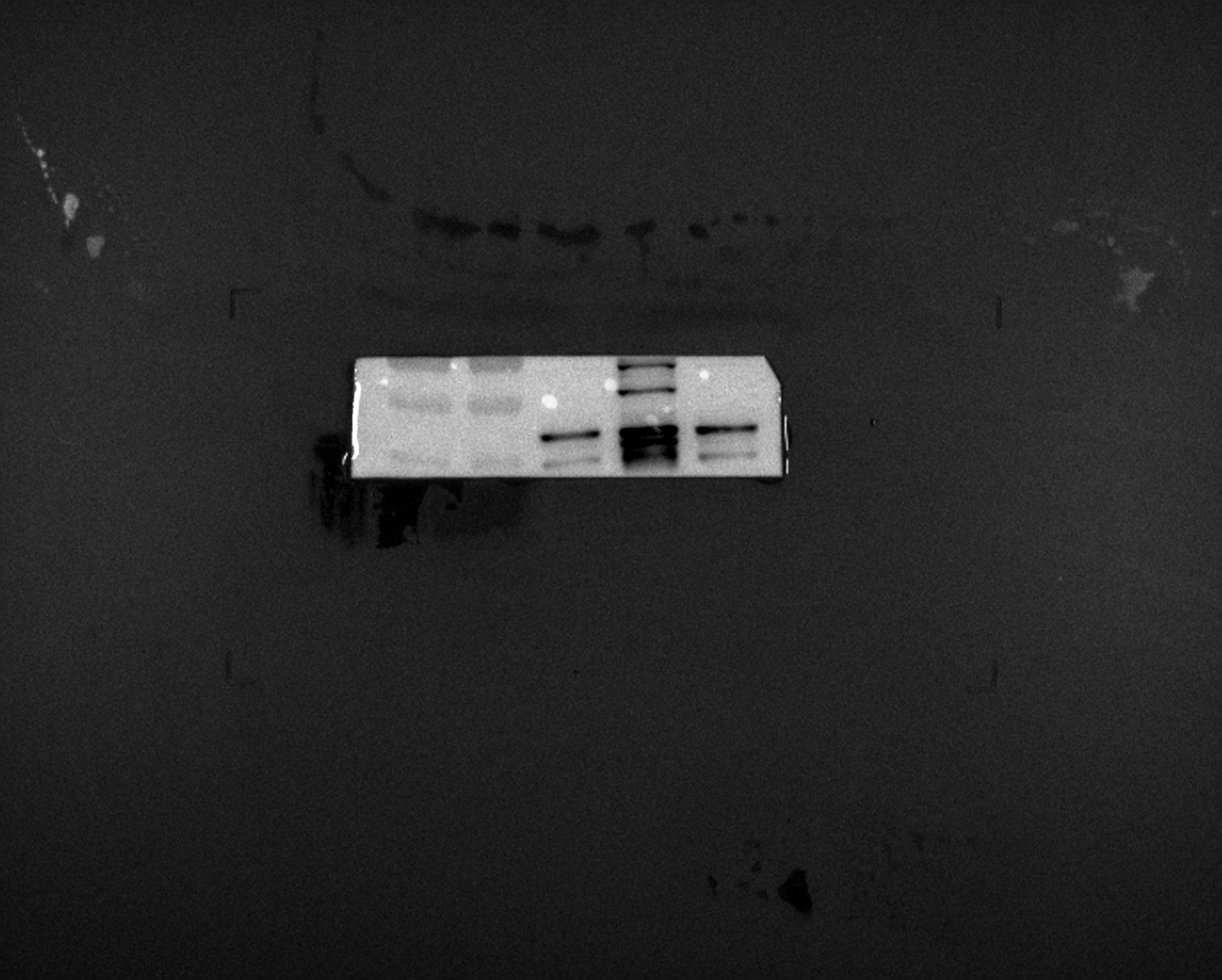

Supplement: Figure 5—source data 4. [file elife-85309-fig5-data4.zip › Figure 5-Source data 4/unedited/G/ALKBH5-merge.tif]

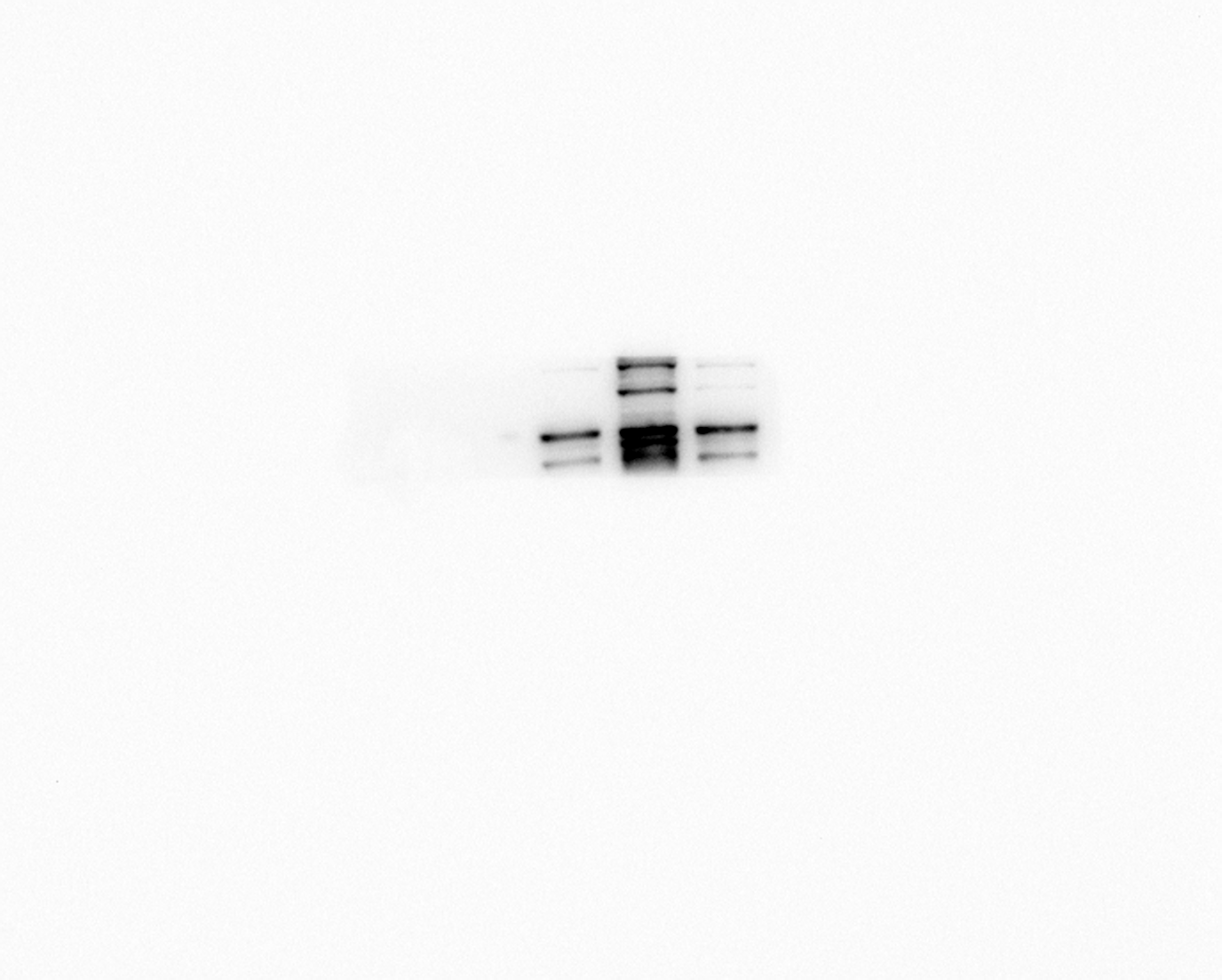

Supplement: Figure 5—source data 4. [file elife-85309-fig5-data4.zip › Figure 5-Source data 4/unedited/G/ALKBH5.Tif]

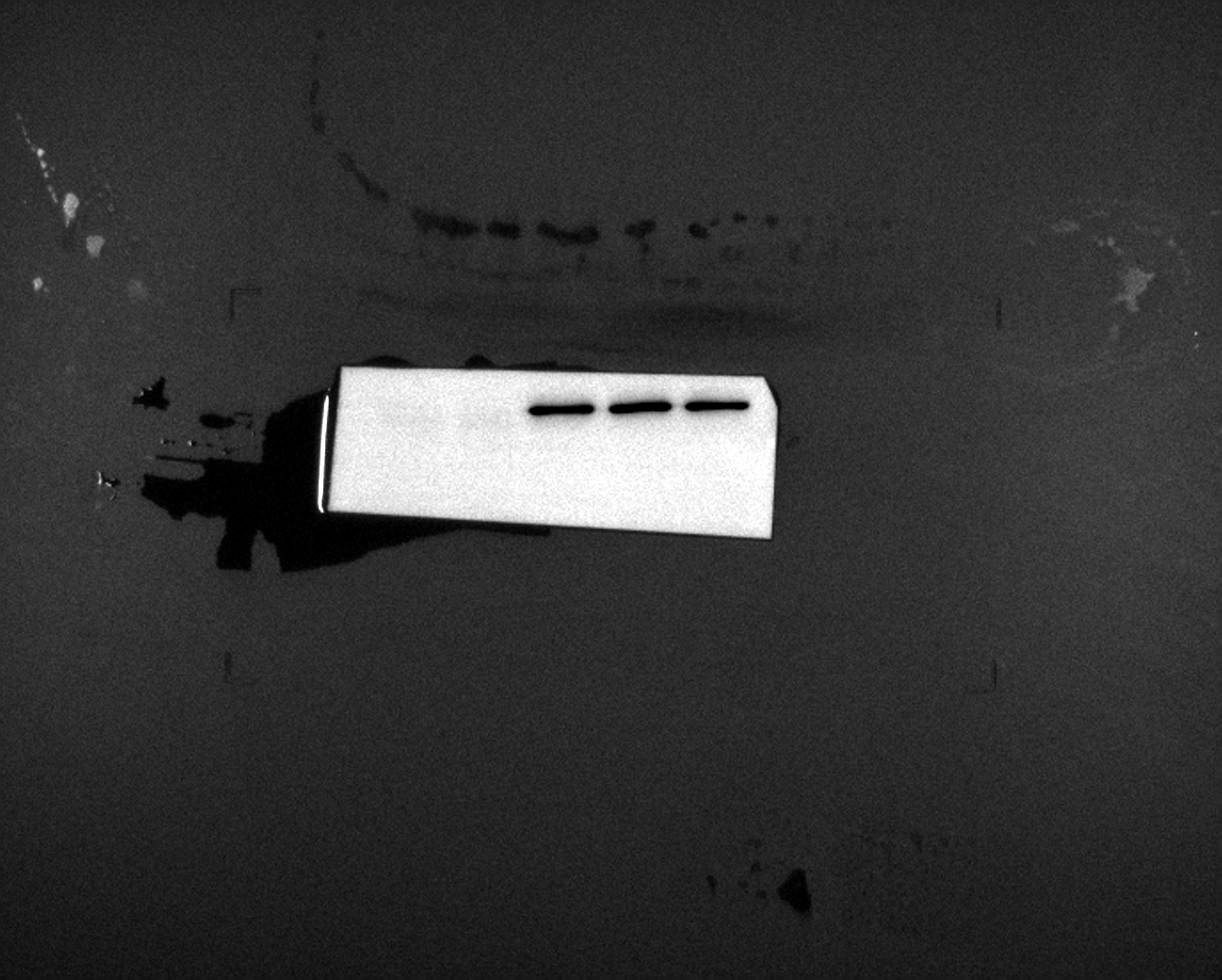

Supplement: Figure 5—source data 4. [file elife-85309-fig5-data4.zip › Figure 5-Source data 4/unedited/G/GAPDH-merge.Tif]

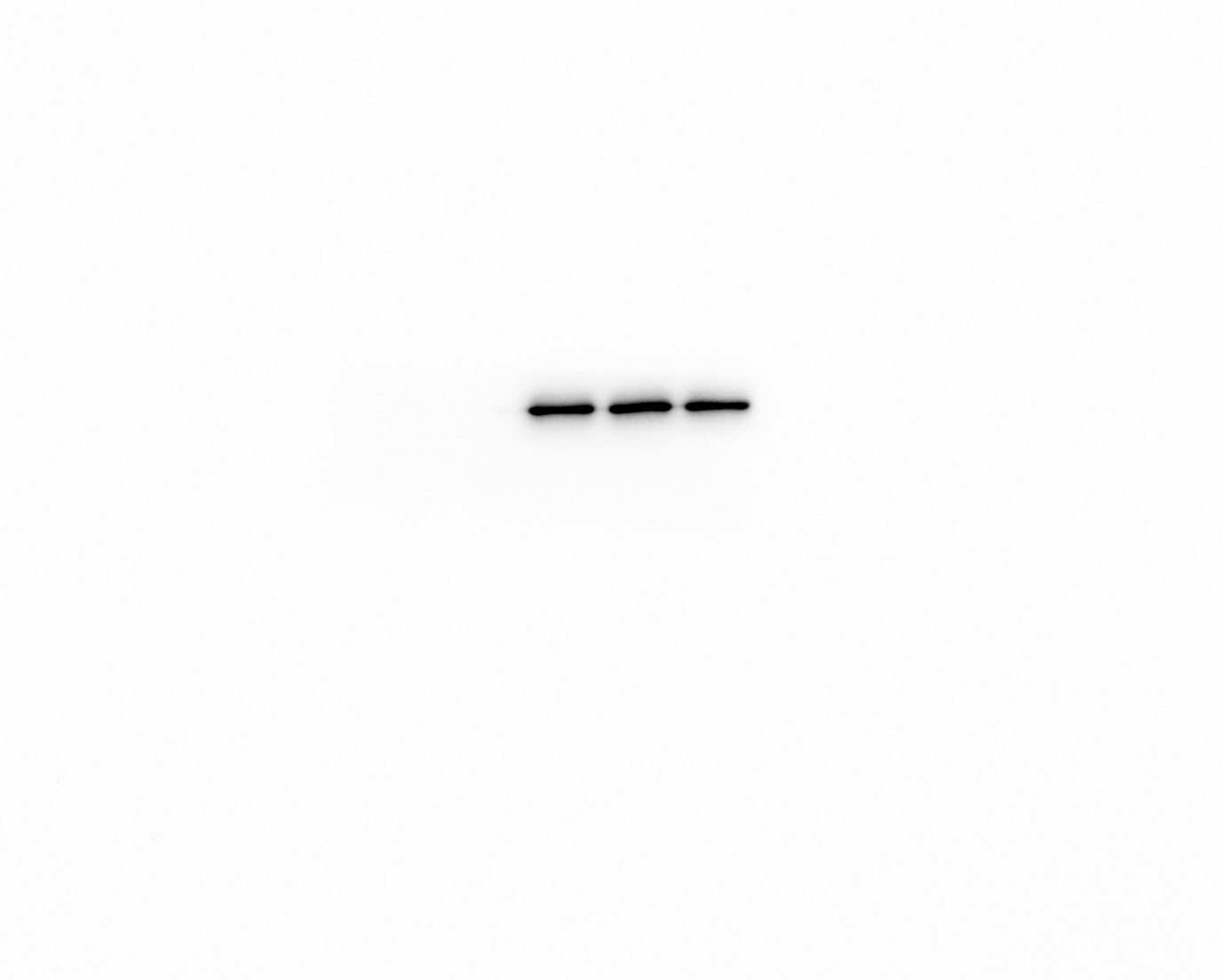

Supplement: Figure 5—source data 4. [file elife-85309-fig5-data4.zip › Figure 5-Source data 4/unedited/G/GAPDH.Tif]

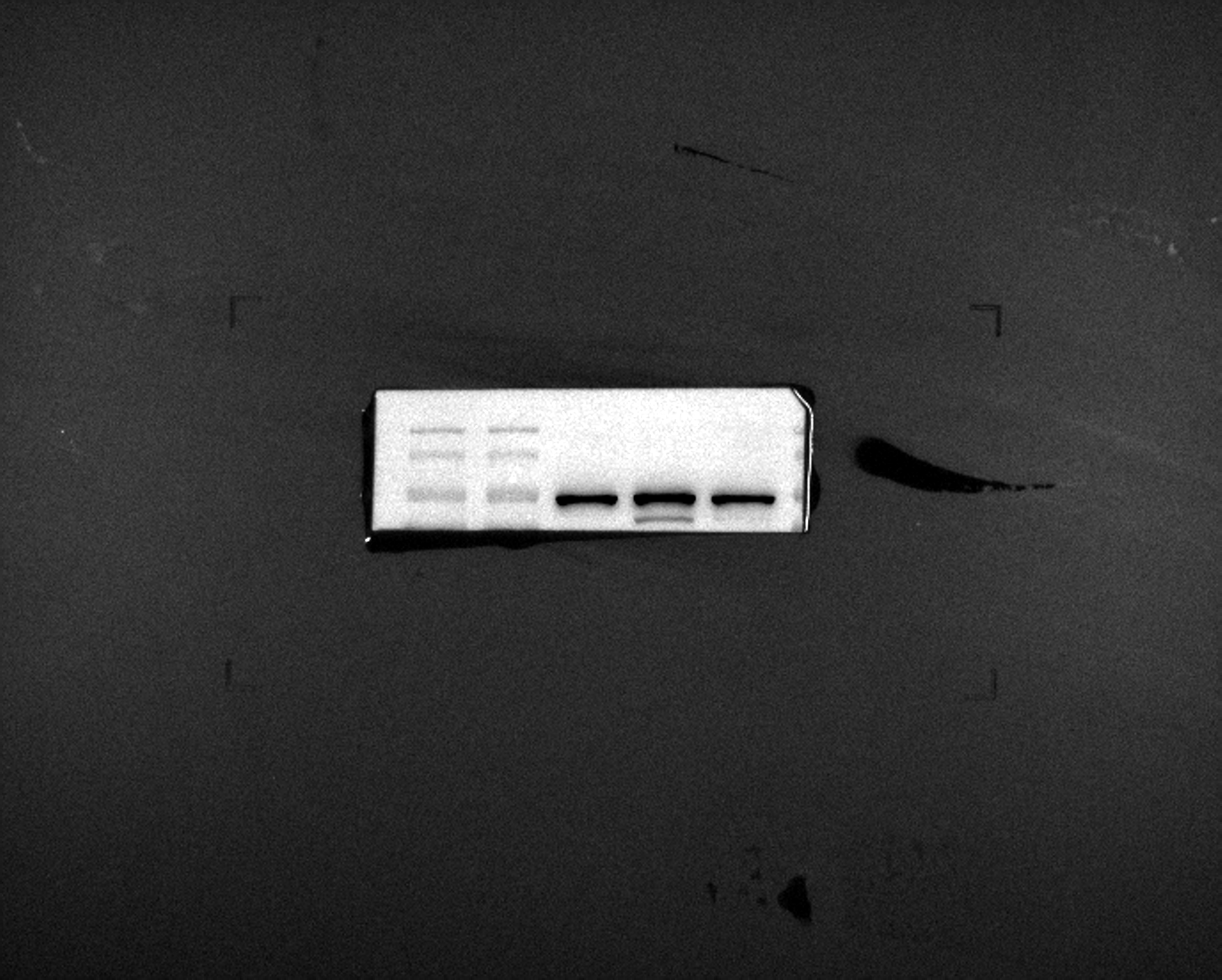

Supplement: Figure 5—source data 4. [file elife-85309-fig5-data4.zip › Figure 5-Source data 4/unedited/G/LPIN2-merge.Tif]

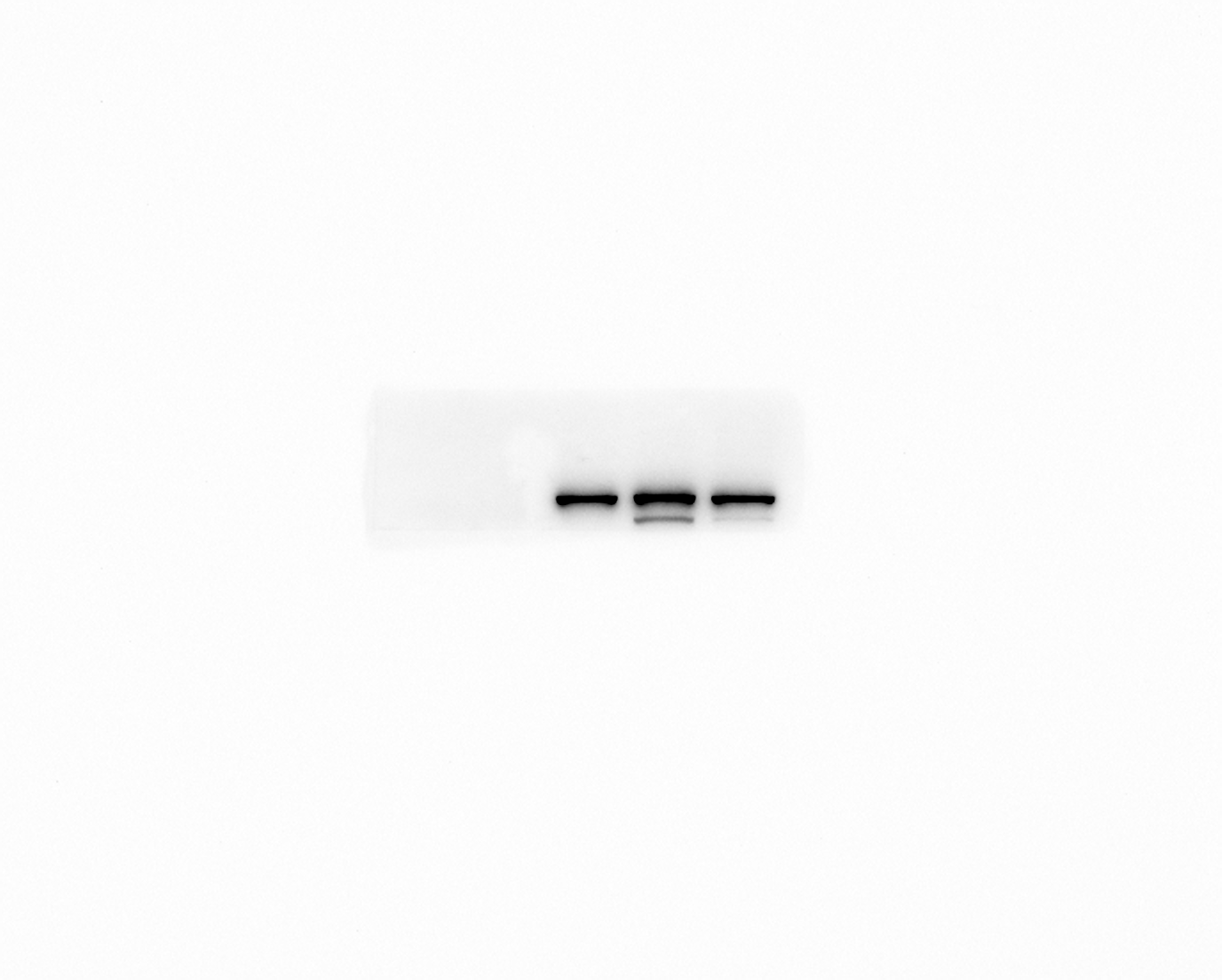

Supplement: Figure 5—source data 4. [file elife-85309-fig5-data4.zip › Figure 5-Source data 4/unedited/G/LPIN2.Tif]

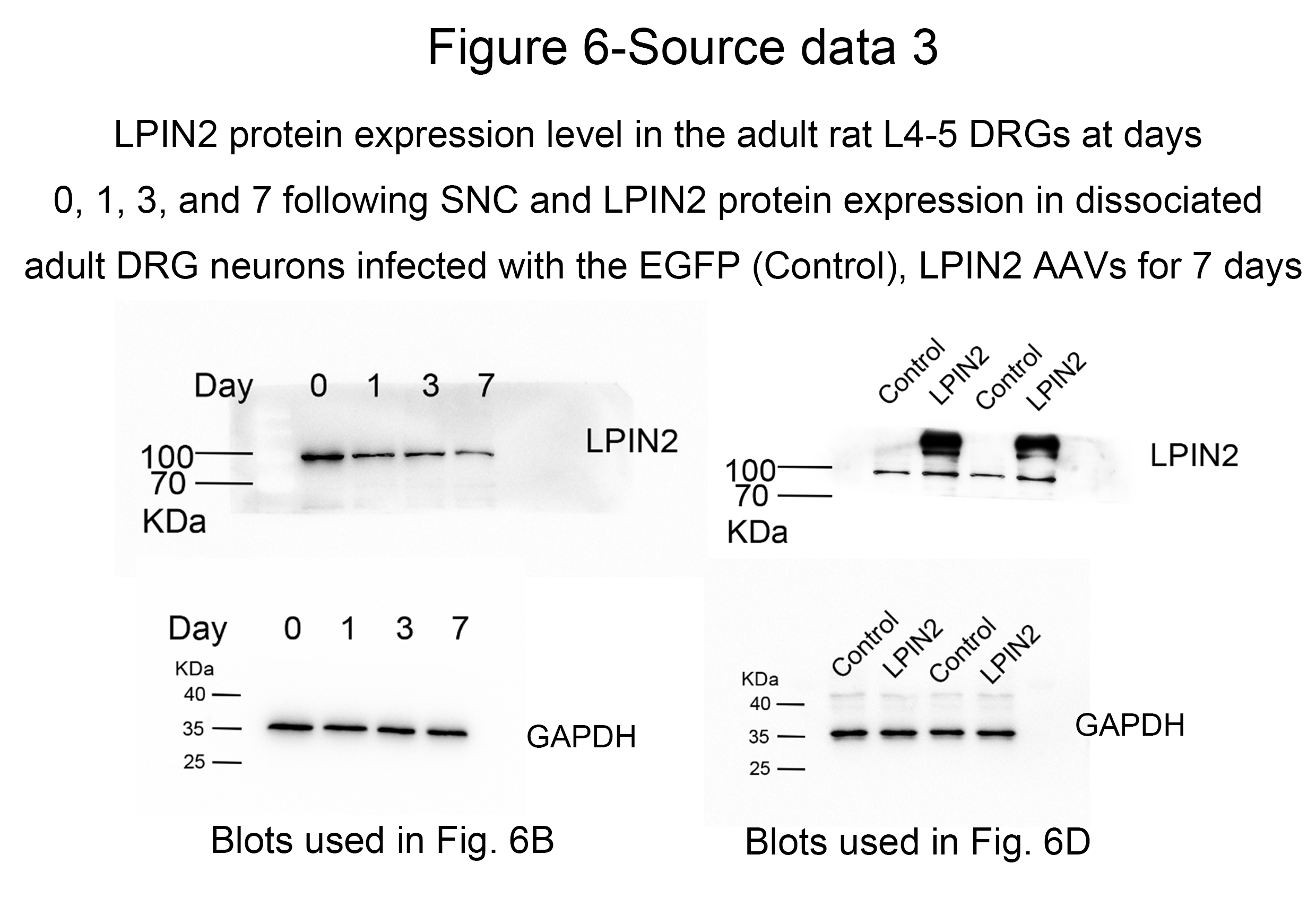

Supplement: Figure 6—source data 3. [file elife-85309-fig6-data3.zip › Figure 6-Source data 3/Figure 6-Source data 3.tif]

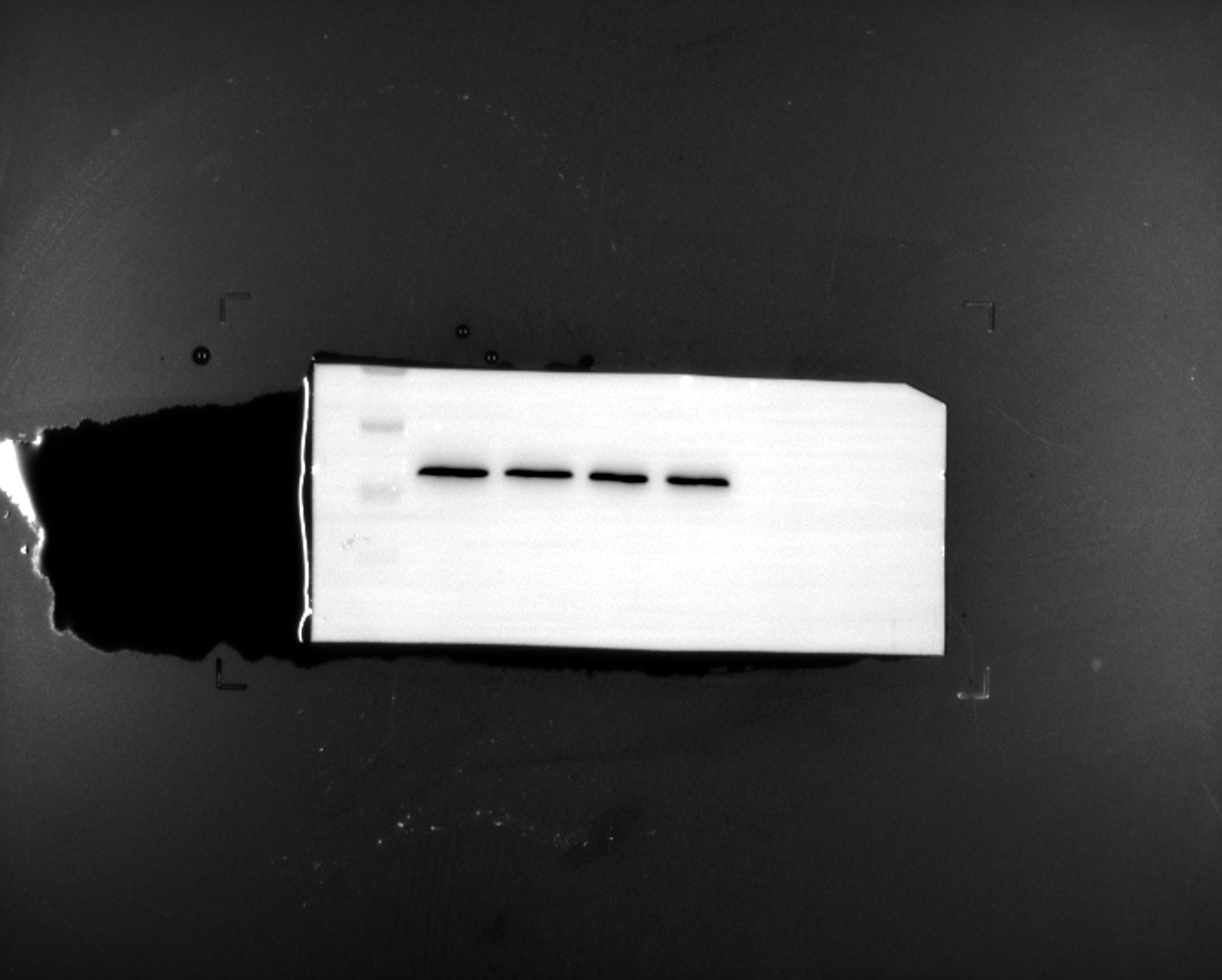

Supplement: Figure 6—source data 3. [file elife-85309-fig6-data3.zip › Figure 6-Source data 3/unedited/B/GAPDH-merge.Tif]

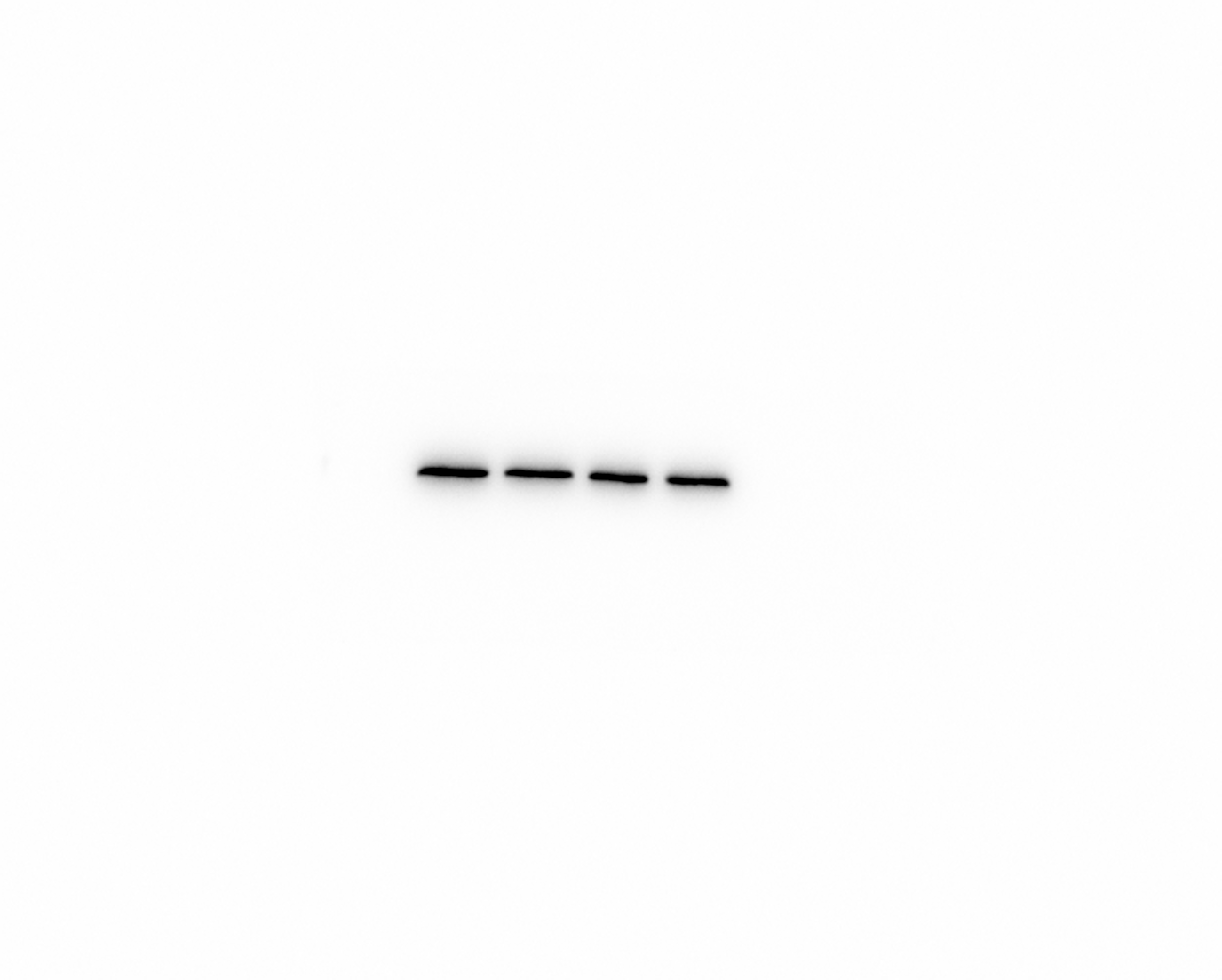

Supplement: Figure 6—source data 3. [file elife-85309-fig6-data3.zip › Figure 6-Source data 3/unedited/B/GAPDH.Tif]

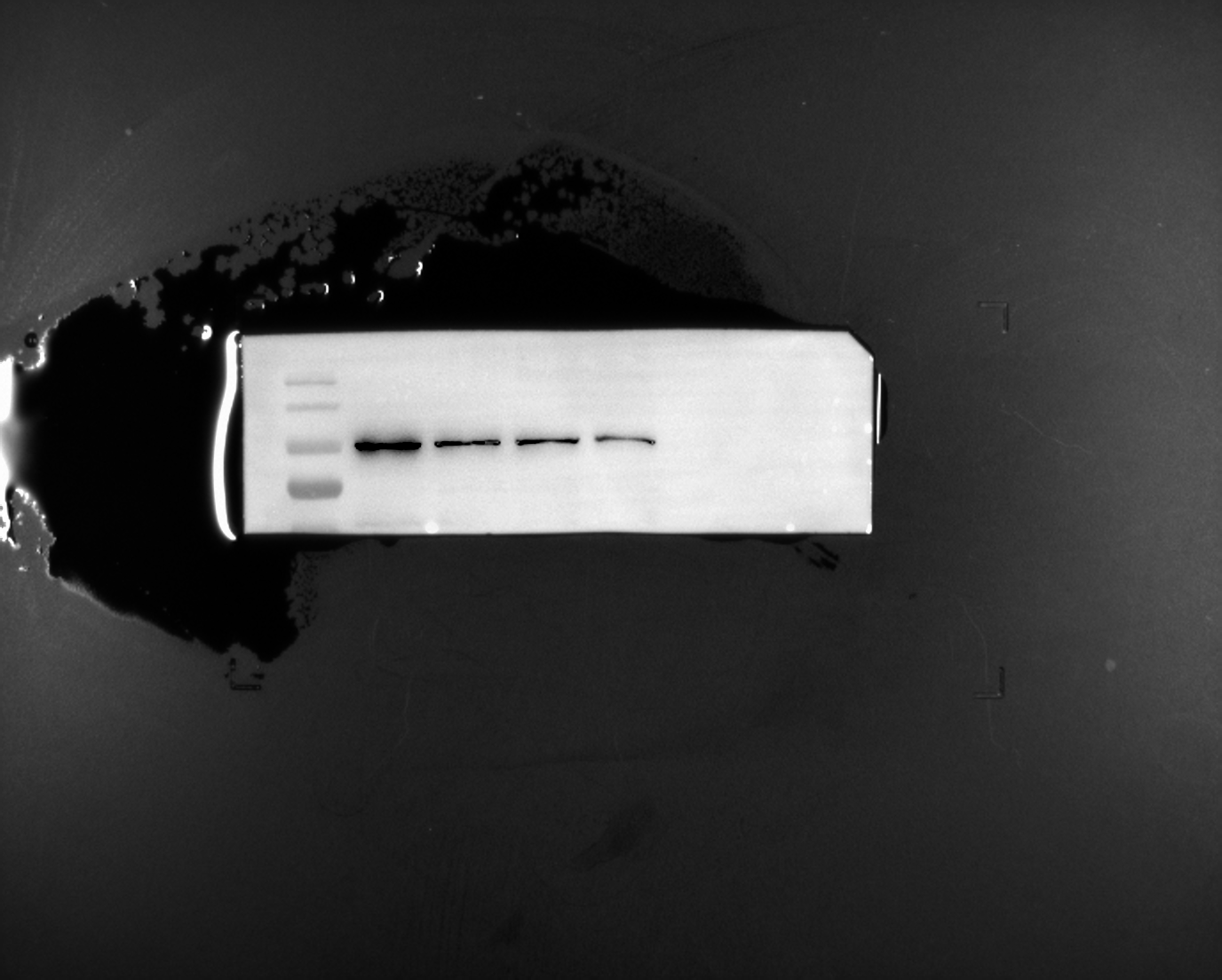

Supplement: Figure 6—source data 3. [file elife-85309-fig6-data3.zip › Figure 6-Source data 3/unedited/B/LPIN2-merge.tif]

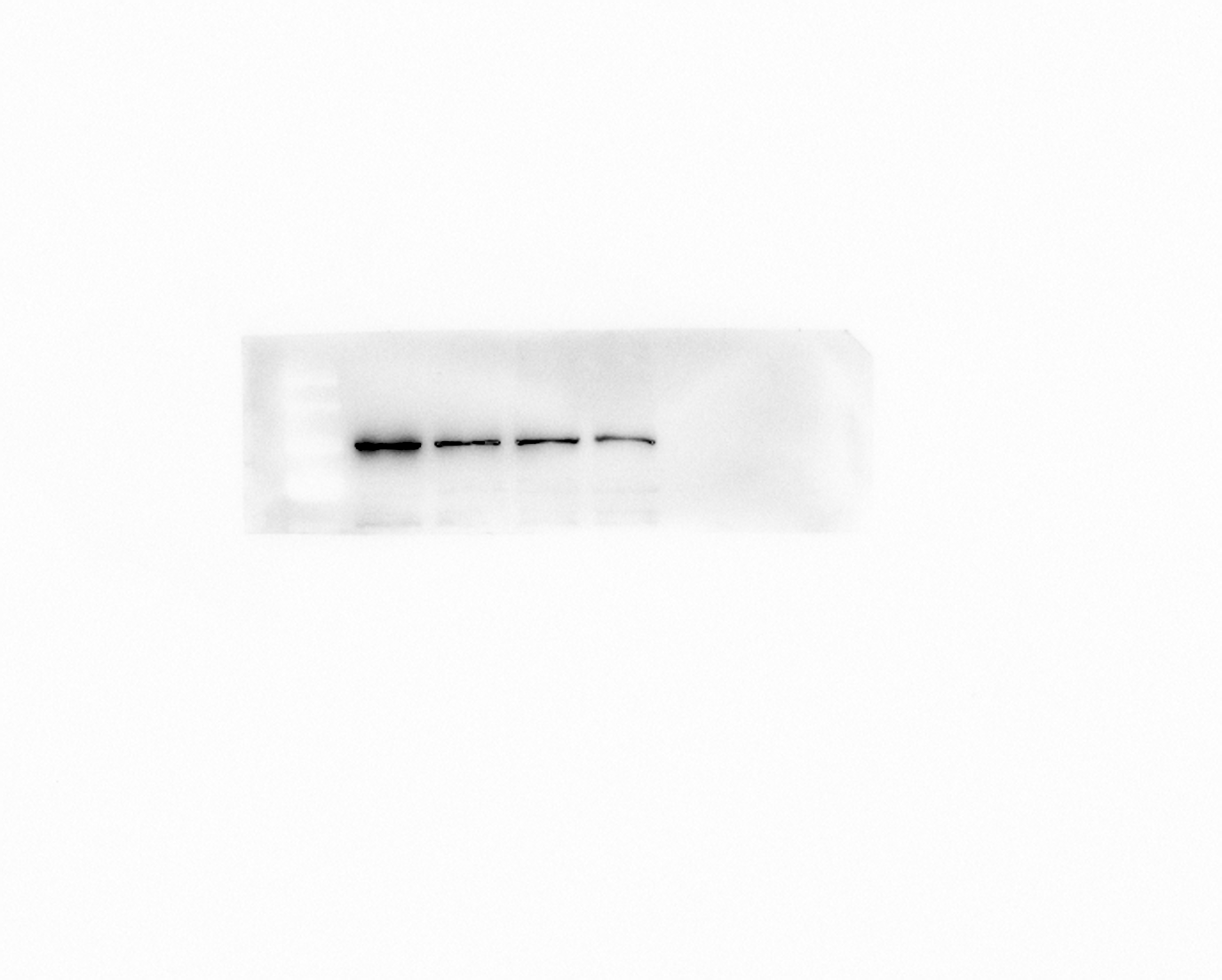

Supplement: Figure 6—source data 3. [file elife-85309-fig6-data3.zip › Figure 6-Source data 3/unedited/B/LPIN2.Tif]

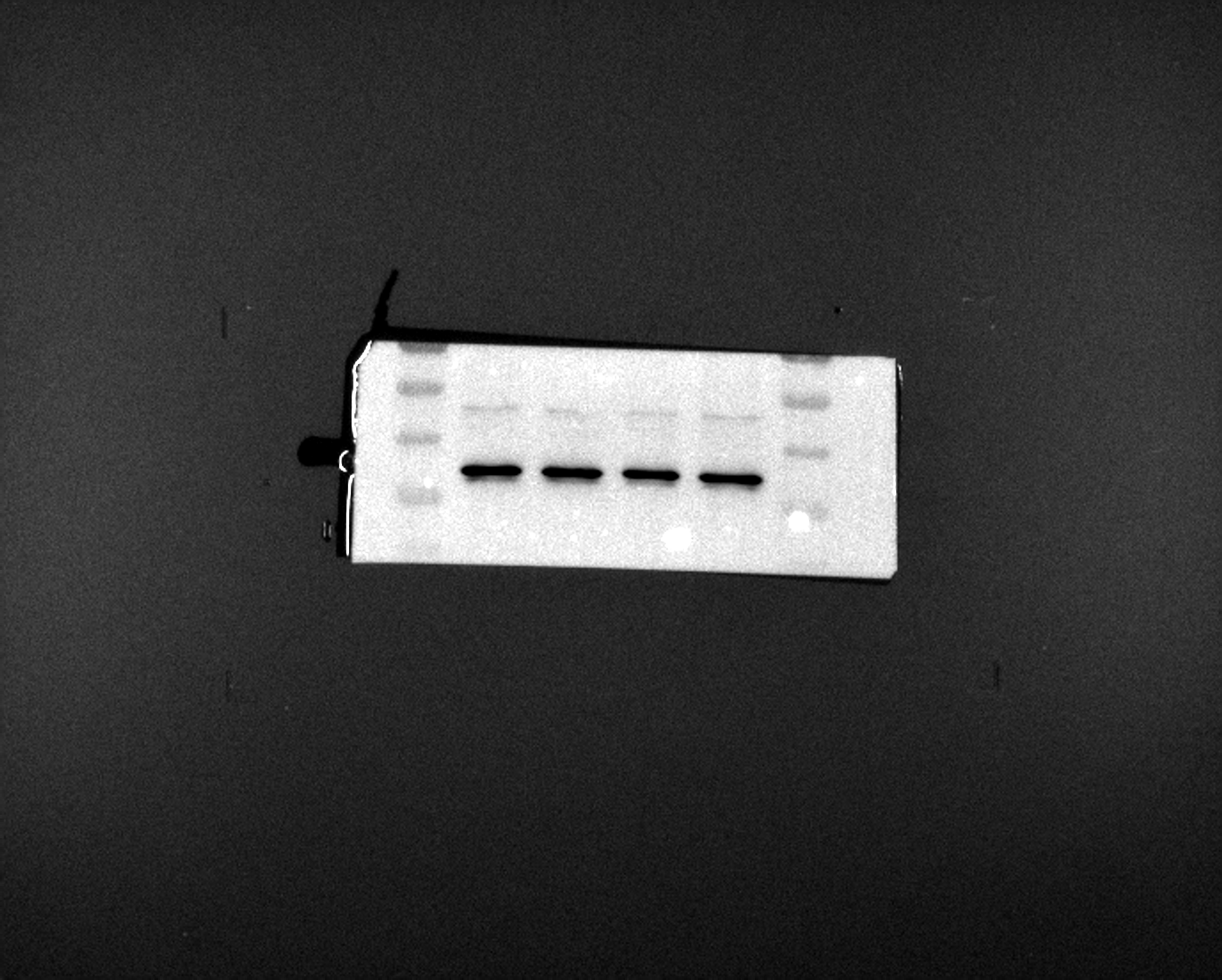

Supplement: Figure 6—source data 3. [file elife-85309-fig6-data3.zip › Figure 6-Source data 3/unedited/D/GAPDH-merge.Tif]

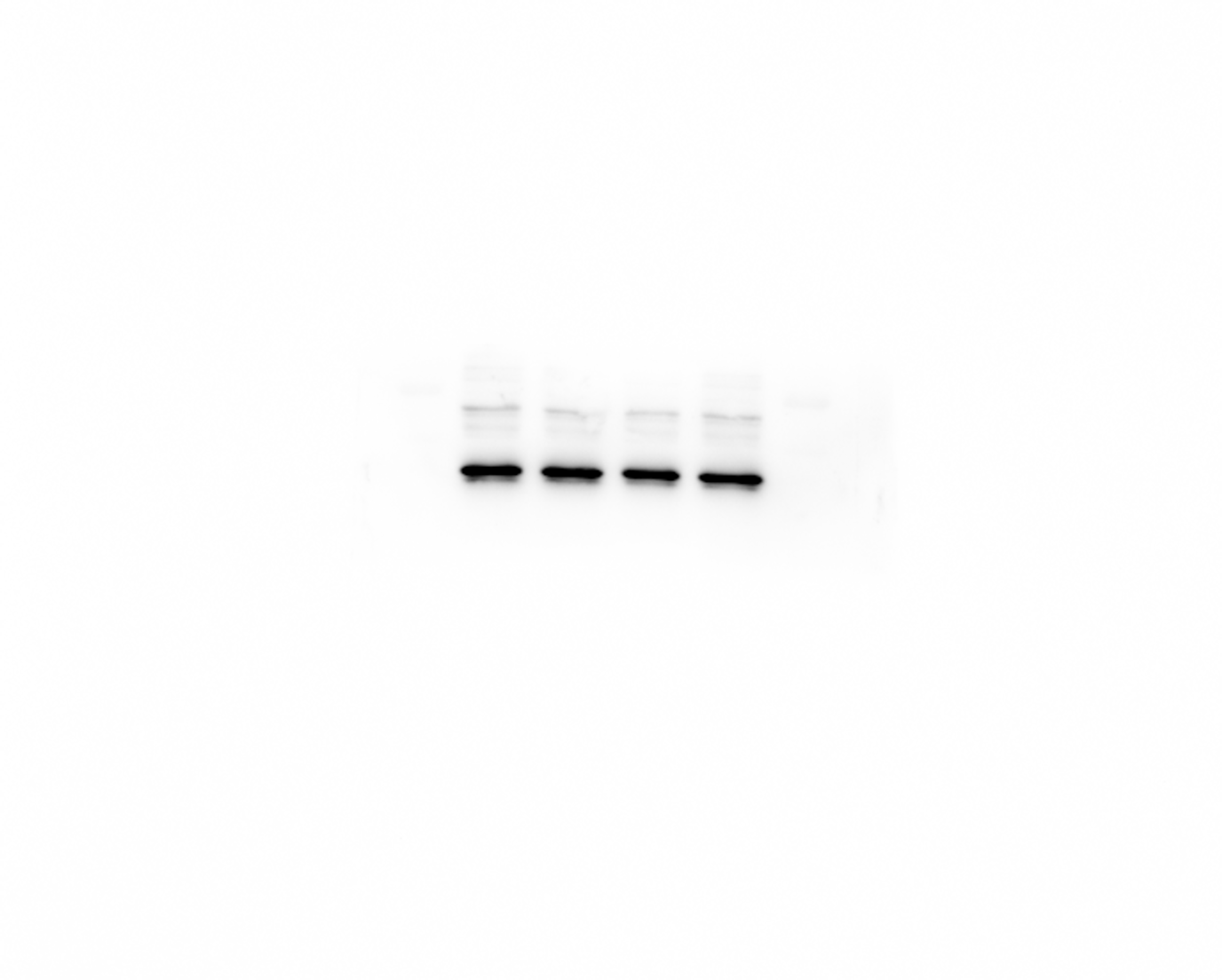

Supplement: Figure 6—source data 3. [file elife-85309-fig6-data3.zip › Figure 6-Source data 3/unedited/D/GAPDH.Tif]

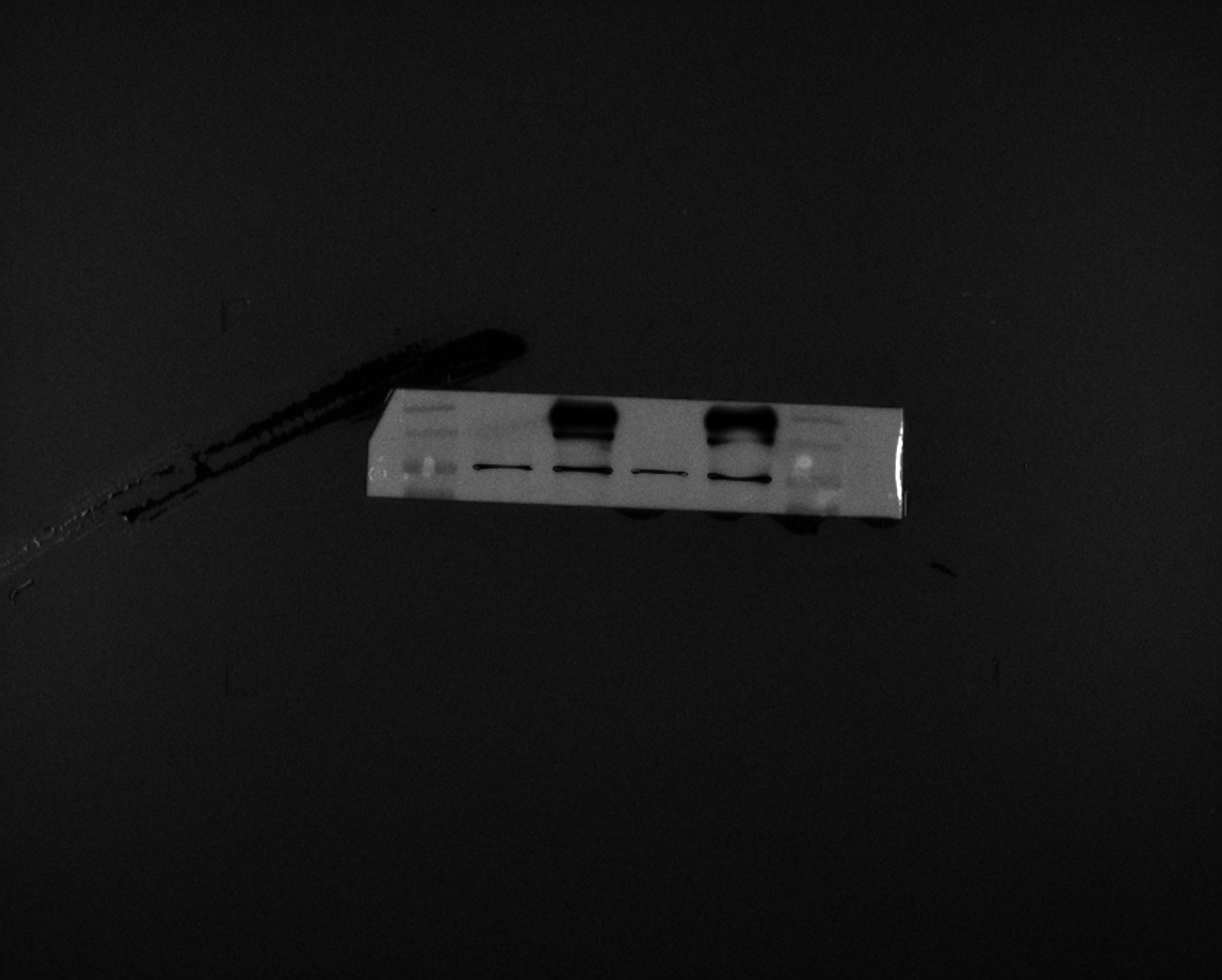

Supplement: Figure 6—source data 3. [file elife-85309-fig6-data3.zip › Figure 6-Source data 3/unedited/D/LPIN2-merge.Tif]

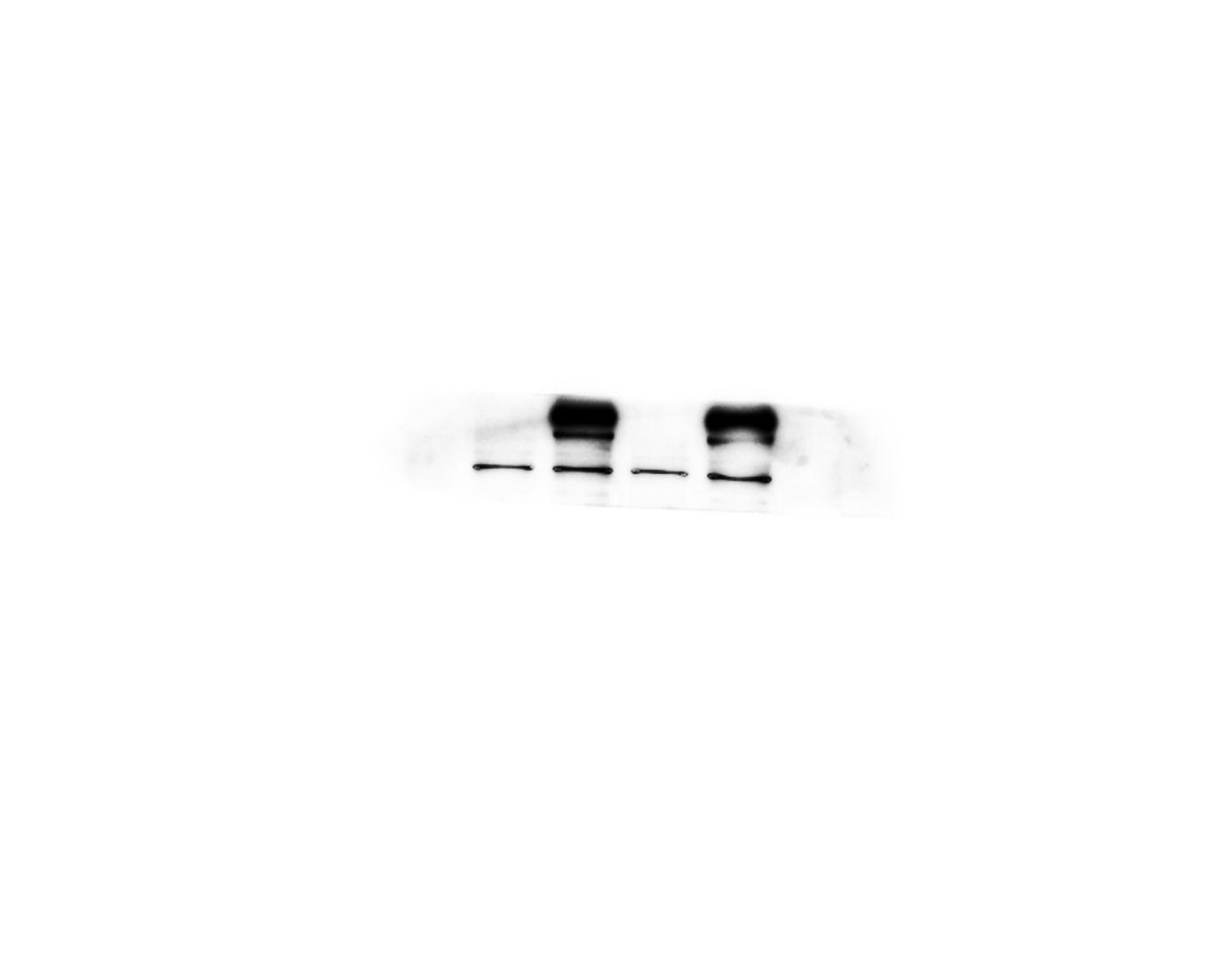

Supplement: Figure 6—source data 3. [file elife-85309-fig6-data3.zip › Figure 6-Source data 3/unedited/D/LPIN2.Tif]
