# Supplementary material for: Promoting axon regeneration by inhibiting RNA N6-methyladenosine demethylase ALKBH5
Source: eLife. 2023 Aug 3;12:e85309. doi: 10.7554/eLife.85309 (PMC10400074; doi:10.7554/eLife.85309)
Supplement: Supplementary file 1. [file elife-85309-supp1.docx]

**Supplementary file 1. Primers for qRT-PCR validation of candidate Genes**

| NO. | Gene | Forward (5’-3’) | Reverse (5’-3’) | Product Size (bp) |
| --- | --- | --- | --- | --- |
| 1 | *Mettl3* | ATGTGCAGCCCAACTGGATT | CTGTGCTTAAACCGGGCAAC | 88 |
| 2 | *Mettl14* | GCTCTGGGGAAGGATTGGAC | AACGGCCTTTGGATCTAGGG | 134 |
| 3 | *Wtap* | CACAGCTTGAAGCCGAGTTG | ATGGTGCTCTGCATACCCTC | 124 |
| 4 | *Fto* | CTGTGTTTTGGCTGGCTCAC | CAAACTCGACCTCGTTGTGG | 209 |
| 5 | *Alkbh5* | GCAAGTTCCAGTTCAAGCCC | CATCAGCAGCATACCCACTGA | 99 |
| 6 | *Ythdc1* | CAGCCGGGAGGAGAAAGATG | GCTTCTGTCGCTTGGTGTCA | 173 |
| 7 | *Ythdc2* | AAAGCAACGAACAGAAAAAGATGT | ACCTGAGCAAAAGCATCGAC | 114 |
| 8 | *Ythdf1* | GACCCCCAGAGAACAAAAGGA | CTGTCCAGAAAGGTAGGGCTC | 105 |
| 9 | *Ythdf2* | CAGGCAAGGCCGAATAATGC | TCTCCGTTGCTCAGTTGTCC | 167 |
| 10 | *Ythdf3* | ACTTTCAAGCACACCACCTCA | TTTCTCTCCCTTCGCATGGC | 89 |
| 11 | *Igf2bp1* | TGAGCAAGTGAACACCGAGA | AGGGCATGGTTCTCCAGTTG | 117 |
| 12 | *Igf2bp2* | ACCCTCTCGGGTAAAGTGGA | TACCGTCCCATACTCAGCCA | 156 |
| 13 | *Igf2bp3* | TTCCTGGTGAAGACGGGCTA | GACCGAGTGCTCAACTTCCA | 126 |
| 14 | *Gapdh* | GGAGAGTGTTTCCTCGTCCC | ATGAAGGGGTCGTTGATGGC | 136 |
| 15 | *Cyp27a1* | GGCACCTTTCCTGAGCTGAT | CTTCCCGAAGGGTACCACAC | 141 |
| 16 | *Aldh3b1* | CTTCAATGCCGGGAGGACGA | CGGACACCTCCGACTCAAAG | 146 |
| 17 | *Aldh3a1* | TCCTCTGTGACCCCTCGATT | CTTTGACCCGCTGGAAGTGA | 138 |
| 18 | *Galns* | TGTGCTACTGCTCATGGACG | TGGGGTCTCTCTGGAAGGTT | 73 |
| 19 | *Fuk* | GGACCAAGTGAGTGGCCTAA | GCCATAAGCTTCCTCTGGACA | 123 |
| 20 | *Ndufa11* | AGTGTCATCGCAAGGCCTAC | GCTGCAGTGAATGTGAACCG | 142 |
| 21 | *Mgat5b* | CAACAGCCAAGATGCGTTCC | AAGGCGGGGTACAAGTGATG | 81 |
| 22 | *Sec1* | CTGGGCTGCCTATCTCACAG | CCTCTGTTTGGCTGTTCCAGA | 150 |
| 23 | *Nmrk1* | ACCCTCCAGGGTACTTCGAT | GACCTTGTTCCGTCCAGGTA | 106 |
| 24 | *Lpin2* | GACTGGACTCATCAGGGCAT | GGCAAGATTGTGCCCTTGTC | 149 |
| 25 | *Pold1* | TCATCTCTCGGGCACAAACC | GGAGGAGTCACGGATGTTGG | 89 |
| 26 | *Amacr* | TGAGTGGATTTGGCCAGTCG | AGGGGAGGGTATGGGTTCTC | 121 |
| 27 | *Cox8a* | TCACTTCCTGCTTCGTGTGT | ACTCCCGCTTCTTGTAGCTTT | 78 |
| 28 | *St6gal1* | ATTCCAGATGCCCAAGAGCC | GGAAGGCTGTGGTTTGACCT | 145 |
| 29 | *Ptgs1* | AACGACAGTACCACCTGCG | ACGCCTGTTCTACGGAAGGT | 93 |
| 30 | *Ndufb7* | CTGAAGTGCAAACGCGACAG | CCGCTCACGTTCAAACTCCT | 120 |
| 31 | *Tpk1* | AGCATGTCTTTACCCCGCTG | AGTGATTGGCACCTCCATCAG | 150 |
| 32 | *St6galnac3* | GTACTGCGACGGGGTGTTTA | GGATACTGTAGCAGGCGTCC | 116 |
| 33 | Pre*-Lpin2* | GATAAGGACCAAGACAGGGCA | CTCAGGGTCCAGACACAAACA | 113 |
