# Supplementary material for: Promoting axon regeneration by inhibiting RNA N6-methyladenosine demethylase ALKBH5
Source: eLife. 2023 Aug 3;12:e85309. doi: 10.7554/eLife.85309 (PMC10400074; doi:10.7554/eLife.85309)
Supplement: Supplementary file 2. [file elife-85309-supp2.docx]

**Supplementary file 2. Interference sequence (IS) designed for target gene**

| Target Gene | IS NO. | Target Sequence (5’-3’) |
| --- | --- | --- |
| *Mettl3* | siRNA1 | GTCTATAGTCCCTGAATTA |
| *Mettl3* | siRNA2 | CCTACAAGATGACGCACAT |
| *Wtap* | siRNA1 | GCGAAGTGTCGAATGCTTA |
| *Wtap* | siRNA2 | GCTGTCCCAGGGCCGTATT |
| *Fto* | siRNA1 | GCTTGAAGACACTTGGCTT |
| *Fto* | siRNA2 | GGACCTGGTGAGGATCCAA |
| *Alkbh5* | siRNA1 | GCCTCAGGACATCAAAGAA |
| *Alkbh5* | siRNA2 | GGATCCTGGAAATGGACAA |
| *Ythdc1* | siRNA1 | GCCAGATTGTCATCAGAAT |
| *Ythdc1* | siRNA2 | CCAGAGACCAGGGTATTTA |
| *Ythdf1* | siRNA1 | CCTATGGACAACTTAGTAA |
| *Ythdf1* | siRNA2 | GGAAGTGCTCAGCCTACTT |
| *Ythdf2* | siRNA1 | GCAGTGGGTTCGGTCATAA |
| *Ythdf2* | siRNA2 | CCACCGTTCCATTAAGTAT |
| *Ythdf3* | siRNA1 | GCGTGGATCAGAGACCTAA |
| *Ythdf3* | siRNA2 | CCTATGGACAAATGAGTAA |
| *Aldh3b1* | siRNA1 | GGACTCGTTTGAAGATAAG |
| *Aldh3b1* | siRNA2 | TCCTCAGAGACAACAAACA |
| *Lpin2* | siRNA1 | AGCACATCATCACGTACCA |
| *Lpin2* | siRNA2 | CACATGAAATTGGGTGATA |
| *Galns* | siRNA1 | GGTGTGTATGGAGAACCTT |
| *Galns* | siRNA2 | GGAAGCTAACCTCACCCAA |
| NC | siRNA | GGCTCTAGAAAAGCCTATGC |
